
JOURNAL INFORMATION
==============================
NLM Title Abbreviation: J Child Orthop
Journal ID: J Child Orthop
NLM Journal ID: 101313582

Journal of Children's Orthopaedics

ISSN: 1863-2521
EISSN: 1863-2548
Publisher: SAGE Publications

ARTICLE INFORMATION
==============================
PMCID: PMC4763147
PMID: 26559959
DOI: 10.1007/s11832-015-0701-9
Article ID: 701
Article version: 1
Subjects: Abstracts

29th Annual Meeting of the Children’s Orthopaedics, Frankfurt am Main, 24–25 April 2015

Electronic publication date: 2015 Nov 12
Print publication date: 2016 Feb
Volume: 10
Issue: 1
First page: 57
Last page: 92
Copyright: © The Author(s) 2015
License: This article is distributed under the terms of the Creative Commons Attribution 4.0 International License (http://creativecommons.org/licenses/by/4.0/), which permits unrestricted use, distribution, and reproduction in any medium, provided you give appropriate credit to the original author(s) and the source, provide a link to the Creative Commons license, and indicate if changes were made.
License URL: https://creativecommons.org/licenses/by/4.0/

issue-copyright-statement: © The Author(s) 2016

==============================

### Contents

Axial Deviations ... 59
Electromagnetic Tracking to Improve Accuracy of Femoral Derotation Osteotomy ... 59
Gait Deviations do not Match Clinical Observations in Patients with Increased Femoral Torsion and Healthy Subjects ... 59
Guiding Growth in Fibular Hemimelia; Rebound of Valgus Deformity Following Temporary Hemiepiphyseodesis ... 60
Description and Evaluation of Operative Deformity Correction in Calcium-Deficiency Rickets in North Nigeria ... 60
Guiding Growth in Preschooler is Safe ... 61
Comparison of FlexTack and Eight-Plate for Temporary Hemiepiphysiodesis ... 61
Hip Dysplasia ... 62
Mismatch of the Femoral Head and the Acetabulum After Open Reduction of the Developmental Dislocated Hip: Does the Use of Currently Available Classification Systems of Osteonecrosis Underestimate the Outcome? ... 62
Effect of Surgical Treatment on Gait Performance and Dynamic Knee Joint Loading in Patients with Legg–Calvé–Perthes Disease ... 62
Decreased Anterior Femoral Neck Off-Set" After In Situ Pinning for Slipped Capital Femoral Epiphysis (SCFE): Correlation with Clinical Symptoms and Range of Motion of the Hip Joint ... 63
Results of Percutaneous Musculotendinous Release in Children with Hip Dysplasia Secondary to Cerebral Palsy Aged Under 6 Years ... 63
Subtrochanteric Femur Fracture as Serious Complication After Operative Stabilization of Slipped Capital Femoral Epiphysis: A Reason Not to Operate the Non-Affected Hip? ... 64
Hip Morphology in MPS 1-H Patients After Hematopoietic Stem Cell Transplantation ... 64
Gait Deviations After Perthes Disease in Dependence of the Radiological Outcome ... 65
Best Lectures ... 65
Treatment of Congenital Wry–Neck (Torticollis) by Dreiss' Method and Computer Aided Analysis of the Face Asymmetry ... 65
Criteria for Successful Arm-Prosthetic Supply in Childhood and Adolescence ... 65
Experiences with Clubfoot Therapy in Older Children in Germany ... 66
A Critical View on In-Patient Treated Hip Luxation in the Era of Ultrasound Screening ... 66
Axial Deviations/Limb Lengthening ... 66
Principle, Indication and Midterm Results of the Femoral Intertrochanteric Valgus Osteotomy in Legg–Calvé–Perthes Disease ... 66
Guided Growth of the Infant's Skull: A Pediatric Orthopedic Method? ... 67
Correction of Static Axial Alignment in Children with Knee Varus or Valgus Deformities Through Growth Guidance: Does It Also Correct Dynamic Frontal Plane Moments During Walking? ... 67
Guiding Growth Supports Correction of Equinus in Residual Paediatric Clubfoot ... 68
Lengthening and Correction of Axial Deviations in Children 68
Limb Lengthening by External Fixation Techniques in 41 Patients Affected by Proximal Femoral Focal Deficiency and Fibular Hemimelia ... 68
The Correction in Complex Deformities of the Femur as in Proximal Focal Femur Deficiency (PFFD) Performing Double Osteotomy of the Femur and Stabilization with an External Fixator ... 69
Disturbed Growth of Spine/Deformities of the Feet ... 69
3-D-Gait Analysis and 4-D-Body-Surface Changes in the Effect of Hypercorrection Braces ("Mirror-Braces") in Left Lumbar Scoliosis (LENKE Type V/VI AIS) ... 69
Rib to Pelvis VEPTR Construct Treatment Duplicates Normal Height Gain of Lumbar Vertebral Growth in Neuromuscular Scoliosis ... 69
Increased Risk of Pathological Fractures Due to Vitamin D Deficiency in Handicapped Adolescent Patients: Clinical Features, Diagnostic and Substitution ... 70
Radiologic Results and Quality of Life Following Scoliosis Surgery in Patients with Severe Cerebral Palsy ... 70
Ventral Epiphyseodesis of the Distal Tibia for Treatment of Limitation of Passive Dorsalextension ... 71
Hallux Varus Congenitus and Hallux Varus Duplicatus ... 71

Surgical Treatment of Rigid Cavovarus Deformity in Children and Adolescents ... 71
Lengthening in Brachymetatarsia with an Internal Fixator ... 72
Correction of Adduction and Supination Residual Deformity in Clubfoot ... 72
Infantile Tumour Surgery/Varia ... 73
The Delayed Diagnosis in Osteo- und Ewing Sarcoma: A Facebook-Based Survey ... 73
Primary Malignant Bone and Soft Tissue Sarcoma in Children and Adolescent: An Analysis of a Nationwide Sarcoma Center ... 73
Influence of Pathological Fractures on the Prognosis of Primary Malignant Bone Tumors ... 73
Morbus Trevor: Clinical Manifestation and Therapy of Dysplasia Epiphysealis Hemimelica ... 74
Osteomyelitis of the Patella: Rare Disease and Therefore Important Not to be Missed ... 74
Poster Presentation ... 74
3 Entities: 1 Goal: Temporary Hemi-Epiphyseodesis to Stop Progression of Severe Varus Deformity. Reports and Review of the Literature for Morbus Blount, Multiple Epiphyseal Dysplasia and Focal Fibrocartilaginous Dysplasia ... 74
3-Level en Bloc Spondylectomy T4-6 of a Spinal NOS Sarcoma G2 in a 7 Year Old Boy ... 75
A Limping Adolescent: Der Intressante Fall ... 75
Age-Dependent 3D Foot Analysis During Heel-Raise of Paediatric Flatfeet ... 76
Altered Rotational Leg Alignment in Flatfeet Predisposes for Knee Overloadings ... 76
Analysis of the Quality of Life in Children with Longitudinal Deficiency of the Lower Extremities, Arthrogryposis Multiplex Congenita or Skeletal Dysplasia ... 77
Anatomic Reconstruction of the Medial Patellofemoral Ligament in Children and Adolescents Using a Pedicled Quadriceps Tendon Graft ... 77
Anterior Knee Pain in a 15 Year Old Girl with a Hyperplastic Trochlear Groove and Femoral Neck Rotation Deformity ... 78
Arthroscopic Reduction and Primary Osteoplasty for Severe and Moderate Slipped Capital Femoral Epiphysis: Surgical Technique ... 78
Biomechanical Evaluation of Two Tension-Band-Principle Based Tools for Guided Growth: FlexTack vs. Eight-Plate ... 78
Camptodactyly-Arthropathy-Coxa Vara-Pericarditis (CACP): Is There a Right Time for Surgical Treatment? ... 79
Correction of Gait After Derotation Osteotomies in Cerebral Palsy: Are The Effects Predictable? ... 79
Correction of Procurvation Deformity near the Knee Joint in Children by Means of Guiding Growth ... 79
Correction of Recurrent Equinus Deformity in Surgical Treated Clubfeet by Anterior Distal Tibia Epiphyseodesis ... 80
Deformity Correction and Lengthening of the Lower Limb, 45 Surgeries in 24 Patients with Various Skeletal-Dysplasia ... 80
Delayed Severe Case of Perthes Disease ... 81
Different Gait Patterns in Patients with Femoral Rotational Malalignment: Influence of Gait Analysis on Treatment Decisions ... 81
Differential Therapy in Infants with Positional Skull Deformity ... 81
Disabling Muscular Weakness and Massive Genua Valga Due to Rickets Secondary to Coeliac Disease ... 82
Femoral Torsion in Children with PFFD (Proximal Focal Femur Deficiency) ... 82
Gait Changes in Boys with Duchenne Muscular Dystrophy After Rideau's Multilevel Soft Tissue Release ... 82
How to Treat-Necrosis of Femoral Head and Neck Caused by Infantile Septic Coxitis ... 83
Hyperthyreosis: Uncommon Reason for Gait Disturbance in Orthopaedic Practice ... 83
If the Nutcracker Becomes a Brainteaser: Case Presentation of a Cuboid Fracture and Lisfranc Joint Injury in a 6 Year Old Boy ... 83
Kniest Dysplasia ... 84
Large Osteochondromas of the Femoral Neck: Results of Resection by an Intertrochanteric Osteotomy ... 84
Magnetic Controlled Growing Rods: First Experience in 12 Patients ... 85
Metamorphosis of Normal Human Lumbar Vertebrae to Quadruped-Like Shape by VEPTR Induced Growth Modulation ... 85
Midfoot Joints Compensate the Decreased Dorsal Extension of Ankle Joint Following Clubfoot Correction ... 85
Mobility Without Wheelchair Despite Complex Dysmorphia-Syndrome ... 86
Multiple Osteochondromata (exostoses) in the Forearm with Carpal Deviation ... 86
Musculoskeletal Manifestations in Mucopolysaccharidosis Type I (Hurler Syndrome) Following Hematopoietic Stem Cell Transplantation ... 86
Postradiation Acetabular Dysplasia in Rhabdomyosarcoma ... 87
Proximal Epiphyseal Slip Following Proximal Varus Derotation Osteotomy of Femur ... 87
Reconstruction of a Traumatic Joint Defect in the Forefoot by Autologous Iliac Apophysis Transplantation in a Child ... 87
Spinal Flexibility in AIS: A Non-Invasive, Pre-Operative and Patient-Specific Method ... 88
The Indication of Genu Valgum Correction by Guided Growth ... 88
The Late Presentation of Knock Knees: A Case Report ... 88
The Results of Preoperative Halo-Gravity Traction for the Treatment of Severe Spinal Deformity in Children ... 89
Treatment of Congenital Pseudarthrosis of the Tibia Using BMP-2: Report of Two Cases ... 89
Treatment of Severe Infantile Blount Disease (Langenskiöld Type 5) in a 5-Year-Old Female: A Case Report ... 90
Treatment of Steroid-Induced Avascular Osteonecrosis Through Core Decompression and Application of Mesenchymal Stromal Cells ... 90
Unilateral Idiopathic Massive Hypertrophy of Intrinsic Foot Muscles ... 91
Valgusdeformity and Malalignement oft the Ankle Caused by Multiple Enchondroma in a Child Suffering From M. Ollier ... 91
Vanishing Bone Disease of the Right Lower Leg ... 91

### Axial Deviations

#### *Axial deviations V1.2*

#### *Electromagnetic Tracking to Improve Accuracy of Femoral Derotation Osteotomy*

T. Dreher*[1], C. Auer[2], M. Niklasch[1], J. Block[2], F. Braatz[3], H. Dickhaus[2], A. Geisbüsch[1]

[1]Universitätsklinik Heidelberg, Klinik für Orthopädie und Unfallchirurgie, Heidelberg, Deutschland, [2]Universitätsklinik Heidelberg, Medizinische Informatik, Heidelberg, Deutschland, [3]Universitätsklinik Göttingen, Orthopädie, Göttingen, Deutschland

***Question*** Rotational abnormalities are common among children with cerebral palsy and treated with femoral derotation osteotomy (FDO). Different authors reported variable outcome including over- or under-correction [1–3]. It could be shown that only 60 % of the derotation performed during FDO can be found in after surgery although K-wires were used to control derotation. The purpose of this study was to introduce an electromagnetic tracking system to improve accuracy of FDO.
***Methodology*** A supracondylar FDO was performed in 13 saw bones and quantified by an instrumental electromagnetic tracking (EMT) hardware used for continuous control of derotation as introduced in [4]. EMT sensor coils were attached to the k-wires applied to the femur proximally and distally to the osteotomy.
Realtime analysis and documentation was carried out using the EMT software.

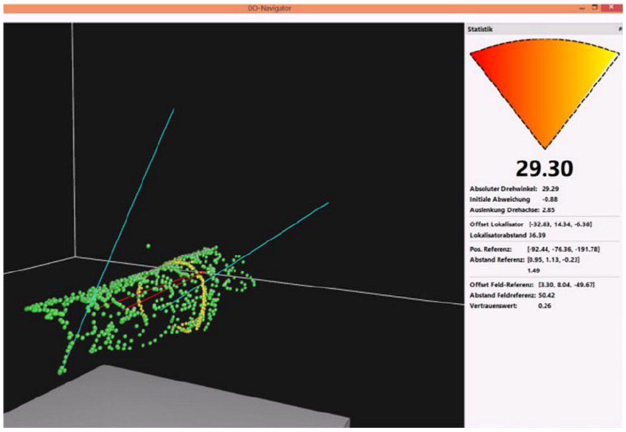

Research software used to process tracking data and monitor derotation in real time

Furthermore 9 different raters measured the rotation amount between the k-wires using a moeltgen goniometer. Standardized measurement of anteversion angle in CT was performed in all saw bones before and after FDO.
***Result*** The derotation amount measured with EMT did not significantly differ (1.2° ± 1.3; p = 0.82) from CT scans. In contrast a significant inter-rater variability was found for goniometer measurement. Furthermore, during fixation of the plate EMT recorded a mean loss of correction (3.8° ± 4.2°).
***Conclusion*** This new EMT system is potent to control correction during FDO reporting the correction with an accuracy ±1° as proven by CT scans. The inter-rater variability of goniometer measurement was high indicating that this method is prone to under- or over-interpretation. Furthermore a relevant change of correction during plate fixation was found by EMT. This may explain variable outcome reported [1–3] underlining the need for a more accurate derotation.

***References***
[1] Kay RM et al (2003) Comparison of proximal and distal rotational femoral osteotomy in children with cerebral palsy. J Pediatr Orthop
[2] Dreher T et al (2007) Internal rotation gait in spastic diplegia–critical considerations for the femoral derotation osteotomy. Gait Posture
[3] Schwartz MH et al (2014) Femoral derotational osteotomy: surgical indications and outcomes in children with cerebral palsy. Gait Posture
[4] Auer C et al (2013) An experimental setup for instrumental analysis of femoral derotation osteotomy. Biomed Tech (Berl)

#### *Axial deviations V1.3*

#### *Gait Deviations do not Match Clinical Observations in Patients with Increased Femoral Torsion and Healthy Subjects*

R. Wegener*[1], F. Wagenblast[2], E. Payne[1], H. Lengnick[1], H. Klima[1]

[1]Children's Hospital of Eastern Switzerland, Department of Paediatric Orthopaedics, Laboratory for Motion Analysis, St. Gallen, Schweiz, [2]The Karlsruhe Institute of Technology, Institute of Sports and Sports Science, Karlsruhe, Deutschland

***Question*** According to the orthopaedic guidelines [1] for the treatment of coxa antetorta, surgical correction during growth is recommended for patients with an antetorsion angle above 50° and fitting complaints. Three-dimensional gait analysis (3DGA) revealed a gait with flexed hips and knees in patients with increased femoral antetorsion (FA). The aim of the study was to examine if these pathological gait patterns measured using 3DGA are in agreement with differences in clinical parameters between groups.
***Methodology*** A 3DGA was performed in 22 patients (mean 12.3 ± 1.8 years) where 13 patients had unilaterally and 9 had bilaterally increased FA (mean 39.2° ± 6.0°) diagnosed with a CT and in a control group of 13 healthy subjects (mean 13.7 ± 2.3 years). For each participant, three gait cycles were retrospectively analysed. Exclusion criteria were age <10 and >18 years, leg length discrepancy >1 cm, foot deformities and neurological diseases. Lower body gait kinematics and kinetics and the range of motion determined in the clinical examination were compared between the patient and control groups using principal component (PC) analysis and linear mixed model. These results were used to qualitatively compare differences between gait analysis and clinical examination in both groups.
***Result*** The clinical examination of hip extension did not differ significantly between the patient and control groups. 3DGA revealed significantly greater tilt of the pelvis (PC1: $p = 0.002$) and hip flexion during the entire gait cycle (PC1: $p < 0.001$) and significantly lower hip extension in pre-swing (PC2: $p = 0.012$) in patients compared to healthy subjects. The popliteal angle was comparable in both groups and patients had significantly greater passive knee hyperextension than the control group (5° vs. 1.1°). Moreover, knee flexion during loading response and terminal stance phase was significantly higher in patients compared to healthy subjects.
***Conclusion*** In addition to the known increased internal hip rotation during walking [2], 3DGA revealed gait deviations typically not detected in clinical evaluations and imaging. These gait deviations possibly lead to structural changes of hip and knee joints while walking with permanent internal hip rotation. Hence, 3DGA facilitates a differentiated decision-making concerning conservative or surgical therapy and should be regarded as an essential diagnostic tool in the orthopaedics guidelines.

***Axial deviations V1.4***

### *Guiding Growth in Fibular Hemimelia; Rebound of Valgus Deformity Following Temporary Hemiepiphyseodesis*

S. Marx*, S. Nader

Schön Klinik, Klinik für Kinderorthopädie, Vogtareuth, Deutschland

***Question*** Fibular hemimelia is a rare congenital defect of the lower limb. Due to hypoplasia or aplasia of the fibular bone the patient reveals numerous deformities of the limb such as shortening of the tibia, knee instability and foot deformity. One of the major problems is the valgus deformity of the knee. Guiding growth with temporary hemiepiphyseodesis of the proximal tibia and/or distal femur is an appropriate and easy way to cope with this deformity and has been applied for many years. Radler et al. examined in 2011 recurrence rates in fibular hemimelia after deformity correction, but did not focus on recurrence after temporary hemiepiphyseodesis. The goal of this study was to reveal the exact recurrence rates of valgus deformity in children with fibular hemimelia depending on the extent of congenital defect, degree of valgus deformity and age at treatment.

***Methodology*** Between January 2009 and January 2015 we monitored 28 patients aged 3–19 years with fibular hemimelia and deformity in the knee which required correction by temporary hemiepiphyseodesis. 26 of these patients could be evaluated for this study. All of them were evaluated with standing a.p. radiographs of the leg before hemiepiphyseodesis, before explantation of 8-plates and at the point of clinically obvious rebound. The X-rays were measured with the TraumaCad program. We documented the development of MAD, the mLDFA, the mMPTA and height of tibial epiphysis before hemiepipyseodesis, at the point of explantation and at the point of clinically obvious rebound.

All patients were treated with hemiepiphyseodesis by 8-plate implantation either only at the proximal tibia and/or distal femur.

***Result*** As we expected, the patients with higher severity of defect such as aplasia showed a higher rate of rebound then the patients with fibular hypoplasia. The younger the child at the point of first implantation, the faster the rebound occurred. The amount of overcorrection did not influence the reoccurrence of deformity itself.

***Conclusion*** Guiding growth by hemiepiphyseodesis is a well established procedure to correct valgus deformity in patients with fibular hemimelia. Patients and parents can be informed prior to treatment that the deformity will reoccur dependent on the age and amount of deformity.

In time of increasing lengthenings with intramedullary nails, this information could also be of a value for obtaining the optimal conditions before such intervention.

***Axial deviations V1.5***

### *Description and Evaluation of Operative Deformity Correction in Calcium-Deficiency Rickets in North Nigeria*

V. Wesselsky*[1], P. Raab[2]

[1]Ev. Waldkrankenhaus Spandau, Abteilung für Orthopädie und Unfallchirurgie, Berlin, Deutschland, [2]König-Ludwig-Haus, Lehrstuhl für Orthopädie der Universität, Würzburg, Deutschland

***Question*** Rickets is a recurrent disease especially in countries with limited resources all around the world. Medical therapy including oral calcium substitution are shown to improve a patients clinical symptoms as well as have an impact on deformities especially in the lower extremity. In a literature review no existing reports about operative deformity correction and its point of intervention in calcium deficiency rickets could be found.

***Methodology*** We describe our concept of operative treatment by subtractive closing wedge osteotomies using mostly k-wires, elastic titan nails and circular plaster cast for osteosynthesis in 45 corrected legs in 27 patients from the rural area of Kaduna, North Nigeria suffering from calcium deficiency rickets. Approximately 10 % of total rickets patients needed orthopaedic intervention and operative therapy after a medical calcium treatment of minimum 2 years and mean age of 12 years with remaining deformities such as Crura valga, Crura vara and tibial antecurvation.

Physical examinations and measurements of angulations and angles were evaluated preoperatively and in a early follow up.

***Result*** We could find a significant improvement in parameters of life quality, functionality, angulation of deformity and angles with a complication rate of 4 % under all osteotomies. In our patient collective suffering from bone softening and immature bone structure, no nonunion or recurrence of deformity could be found.

| | Normal range | Preop | n | Deviation | Postop | n | Deviation |
|---|---|---|---|---|---|---|---|
| TFA valgus | 4°–7° | 39.4° | 28 | 32.4° | 4.8° | 28 | 0° |
| TFA varus–postop varus | (−4°) to (−7°) | 31.2° | 14 | 35.2° | 5.8° | 5 | 9.8° |
| TFA varus–postop valgus | 4° to 7° | | | | 3.3° | 10 | 0.3° |
| LDFA valgus | 79° to 83° | 59.9° | 26 | 19.1° | 86° | 28 | 3° |
| LDFA varus | 79° to 83° | 112° | 8 | 29° | 92° | 8 | 9° |
| MPTA valgus | 85° to 90° | 117° | 2 | 27° | 87.5° | 2 | 0° |
| MPTA varus | 85° to 90° | 64° | 9 | 21° | 85.3° | 9 | 0° |
| Tibial antecurvation | 0° | 79.3° | 7 | 79,3° | 7.3° | 7 | 7.3° |

Angles and angulation

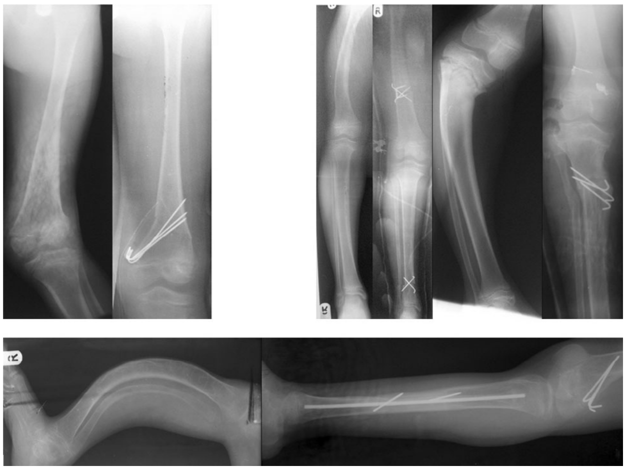

Subtractive closing wedge osteotomies in knockknees, bowlegs and tibial antecurvation

***Conclusion*** The described therapy shows to be sufficient and with satisfactory results in correcting rickets-related leg deformities under rural circumstances. In bowlegs we made the experience that they are significantly more affected with a lateral knee joint laxity than knock-knees are affected with a medial knee joint laxity. Therefore a correction of bowlegs requires a minimal overcorrection into a genu valgum so that the mechanic axis of the leg is securely shifted from the lateral to the medial knee compartment and its more stable ligament structures.

***Axial deviations V1.6***

### *Guiding Growth in Preschooler is Safe*

L. Schwering*[1], V. T. Förster[2]

[1]Mathias-Spital, Technische Orthopädie, Rheine, Deutschland, [2]Universitätsklinikum Freiburg, Department Orthopädie Traumatologie, Freiburg, Deutschland

***Question*** Meanwhile the use of the 8-plate for guiding growth in the correction of axial deformities has become a standard procedure in paediatric orthopaedics. The application of 8-plates in children under the age of 8 years has been seen critically in the German speaking countries. This study shall demonstrate the safeness of this intervention in this age.

***Methodology*** During the years 2005 to 2014 8 girls and 21 boys with a mean age of 4 10/12 years were treated by Stevens's method of guiding growth for the correction of axial deformities in the lower limb (varus or valgus near the knee joint). In particular 37 genua vara and 13 genua valga were operated on and the follow up was observed by clinical and radiographic means. The mean follow-up is 38 months. Complications were evaluated by Paley's score in problems, obstacles, minor and major complications.

***Result*** The mean operation time was 26 min for one deformity. Intraoperative complications were not observed. Postoperative limitations in the range of movement were lost in 1 week after surgical intervention. In all cases a complete correction of the deformity was achieved. In eight patients a recurrence of the deformity were observed because of the primer disease, which led to a second treatment with 8-plates. Finally a complete correction could be reached in this case also. After removal of the hardware, after 10 months in mean, no negative influence was seen on the growth plate.

***Conclusion*** Especially children under the age of 6 will take profit from a guiding growth procedure because the disadvantages of osteotomies like surgical approach and immobilisation can be avoided. This method has proved to be save especially in these patients.

***Axial deviations V1.9***

### *Comparison of FlexTack and Eight-Plate for Temporary Hemiepiphysiodesis*

B. Vogt*[1], M.-T. Kleine-König[1], G. Gosheger[2], H. Tretow[1], M. Horter[1], F. Schiedel[1], R. Rödl[1]

[1]Kinderorthopädie, Deformitätenrekonstruktion und Fußchirurgie, Universitätsklinikum, Münster, Deutschland, [2]Klinik für Allgemeine Orthopädie und Tumororthopädie, Universitätsklinikum, Münster, Deutschland

***Question*** Hemiepiphysiodesis (HED) is the first-line treatment for coronal angular deformities of the knee in growing children. Modern implants like the eight-Plate (EP) solved many problems observed using conventional staples for temporary HED. Rather than creating rigid compression forces on the growth-plate (GP), the EP creates a flexible tension-band effect with extraphyseal fulcrum for guided growth. However, implant design, surgical technique and alignment of the EP remain suboptimal leading to implant-associated and biomechanical problems like delayed onset of correction.

***Methodology*** Therefore a new device for temporary HED was developed that consists of an anatomically preformed titanium staple with cannulated legs ensuring the simple and precise implantation technique of the EP. The 13° trapezoid shaped design properly fits to the anatomic shape of the medial and lateral aspect of the distal femur and proximal tibia. It provides a flexible bar (FlexTack (FT)) that permits the extraphyseal tension-band effect by analogy with the EP. A prospective FT-cohort was compared to a historical EP-cohort. Data are summarized in Table 1.

Table 1

| | | FlexTack | Eight-Plate |
|---|---|---|---|
| Patients/ implants/ Age | Total | 88 (m = 61, f = 27)/ 207/ ~ 11.9 years | 93 (m = 53, f = 40)/ 246/ ~ 11.7 years |
| | Valgus | 57 (m = 34, f = 23)/ 133/ ~ 11.9 years | 56 (m = 31, f = 25)/ 123/ ~ 11.7 years |
| | Varus | 20 (m = 16, f = 4)/ 43/ ~ 12.0 years | 17 (m = 10, f = 7)/ 33/ ~ 11.0 years |
| | Flexion | 2 (m = 2, f = 0)/6/ ~ 11.4 years | 0 (m = 0, f = 0)/0/– |
| | Limb length discrepancy | 9 (m = 9, f = 0)/25/ ~ 12.4 years | 29 (m = 15, f = 14)/ 90/ ~ 12.3 years |
| Follow up | | ~ 6.0 months (max. 1.0 year) | ~ 1.0 year (max. 2.4 years) |

Patient's data

Intraop. parameters like time for surgery (cut-suture) and for fluoroscopy were assessed. Clinical-radiographic follow-up was performed every 3–6 m. Correction speed was analysed in the valgus-group. Complications were evaluated focusing implant-associated and biomechanical problems. Statistics was done by U- and exact-Test.

***Result*** Results are summarized in Table 2.

Table 2

| | FlexTack | Eight-Plate | *P* value |
|---|---|---|---|
| Operation time [min] | 22.4 | 36.8 | <0.001 |
| Fluoroscopy time [min] | 0.26 | 0.42 | <0.001 |
| Correction speed [mm/month/ HED-location] | 3.27 | 1.59 | <0.001 |
| Infection required revision surgery | 1 Patient (1.1 %) | 1 Patient (1.1 %) | 1000 |
| Hematoma/effusion | 5 Patients (5.7 %) | 3 Patients (3.2 %) | 0.488 |
| Neurovascular damage | 0 Patient | 0 Patient | 1.000 |
| Implant-associated complication | 0 Patient | 10 Patients (10.8 %) | 0.006 |

Time for implantation and for fluoroscopy were significantly shorter using the FT. Earlier onset and significantly faster speed of valgus correction were measured for the FT

Common complications were comparable. However, implant-associated problems were not observed using the FT as yet.
Complete corrections were seen in 16 pat., whose 47 FT could be removed without complications.
***Conclusion*** The FT is a reasonable synthesis of staples and cannulated screw-plate-devices. The implantation is simpler which reduces time for surgery and radiation exposure. Due to the anatomical shape and biomechanical improvements faster correction and lower rates of implant-associated problems could be achieved.

### Hip Dysplasia

***Hip dysplasia V2.1***

#### *Mismatch of the Femoral Head and the Acetabulum After Open Reduction of the Developmental Dislocated Hip: Does the Use of Currently Available Classification Systems of Osteonecrosis Underestimate the Outcome?*

R. Pospischill*, R. Ganger, F. Grill

Orthopädisches Spital Wien Speising, Abt. für Kinderorthopädie und Fusschirurgie, Wien, Österreich

***Question*** Several classification systems for the development of osteonecrosis (ON) following treatment of DDH have been reported. Excellent prognosis is attributed to ON grade I as the growth plate remains intact, with little or no changes on the proximal femoral anatomy. ON grade II to IV describe specific femoral head deformities combined with a shortening of the neck.
The purpose of the present study was to find out if open reduction of the developmental dislocated hip could lead to an overgrowth of the femoral head and a lengthening of the neck with radiological changes not corresponding to any published ON grades but resulting in decreased lateral head coverage (mismatch).
***Methodology*** A retrospective cohort study of children with hip dislocation aged less than 12 months at time of reduction, who have been hospitalized at our department between January 1998 and February 2007, was completed. 49 patients satisfied the criteria for inclusion and were treated with closed or open reduction with preliminary traction after initial failed Pavlik harness treatment. The mean patient age at time of reduction was 3.8 months. The average duration of FU for all patients was 7.4 years.
***Result*** For the cohort, the overall rate of ON grade I to IV was 25.8 %. Seven hips (29.2 %) treated with closed reduction were radiologically abnormal and could be assigned to ON grade I to IV. Fourteen hips (56 %) treated with open reduction were radiologically abnormal but only six (24 %) out of 14 (52 %) could be assigned to ON grades. The remaining eight hips (32 %) showed an increased femoral head diameter and neck length compared to the normal side with a decreased lateral head coverage.
***Conclusion*** The femoral part of mismatch in DDH is based on an aspherical femoral head deformity and shortening of the femoral neck resulting from ON after closed or open reduction. Our observation suggest that open reduction could also lead to an overgrowth of the femoral head and lengthening of the femoral neck causing a decreased lateral head coverage. These radiological changes could not be assigned to any ON grades of currently available classification systems. It therefore is reasonable to assume that first, currently available classification systems are not applicable in daily clinical practice. Second, in a critical assessment, published rates of ON are too low and only a minority of hips could be judged as normal. Further research with a large-enough sample size to assess normal hip growth after open reduction is necessary.

***Hip dysplasia V2.2***

#### *Effect of Surgical Treatment on Gait Performance and Dynamic Knee Joint Loading in Patients with Legg–Calvé–Perthes Disease*

F. Stief*[1], S. Adolf[2], L. Kremer[2], A. Schmidt[3], M. Brkic[2], A. Meurer[2]

[1]Orthopaedic University Hospital Friedrichsheim gGmbH, Movement Analysis Lab, Frankfurt/Main, Deutschland, [2]Orthopaedic University Hospital Friedrichsheim gGmbH, Frankfurt/Main, Deutschland, [3]Johann Wolfgang Goethe University, Frankfurt Initiative for Regenerative Medicine, Frankfurt/Main, Deutschland

***Question*** Current outcome evaluations of containment improving surgery in patients with Legg–Calvé–Perthes disease (LCPD) are based on standard clinical parameters and radiological changes. However, previous results showed that the physiological knee joint loading during walking is preoperatively shifted into a pathological valgus moment [Stief et al. (2014) Gait Posture 39:859–64]. The aim of the present study was to evaluate the outcome of containment improving surgery based on selected gait parameters. In particular, it is still not known if the preoperative existing dynamic valgus thrust of the knee can be normalised.
***Methodology*** Sixteen children [14 male, 2 female; mean age of 6.1 (±1.7) years] with unilateral diagnosis of LCPD and 19 healthy subjects (14 male, 5 female) at the same age participated in this prospective longitudinal study. Patients were treated with femoral varus osteotomy, isolated or in combination with pelvic (Salter or Triple) osteotomy. Instrumented gait analysis was performed before and 13.4 (±2.5) months after surgery.
***Result*** Postoperatively, the mean leg length of the involved side was reduced by 0.6 (±0.8) cm compared to the non-involved side. The knee flexion during stance phase was still reduced after surgery compared to the control group ($p < 0.001$). The external knee adduction moment, which is a parameter for characterizing intrinsic compressive load in the knee joint, was postoperatively still different in comparison to healthy subjects at the same age ($p = 0.049$).
***Conclusion*** Despite predominantly good results shown by the standard clinical and radiographic examinations for the hip joint, gait analysis detected various functional deficits at the level of the knee after surgery. Patients displayed stiff knees on the affected side, probably to compensate for leg length discrepancy. This altered gait pattern might be one explanation for the valgus thrust of the knee, which could be sufficient to deform the lateral compartment or influence the remaining growth plate and the physiological development of the mechanical axis of the leg in young patients with LCPD. A further explanation for this pathological knee joint loading may be the result of the surgery due to a change of the coronal plane alignment of the proximal femur. In conclusion, lower limb alignment and dynamic knee joint loading should be controlled during the process of the disease and the postoperative rehabilitation period to avoid degenerative changes in the lateral knee compartment in young patients with LCPD.

***Hip dysplasia V2.3***

### *Decreased Anterior Femoral Neck Off-Set" After In Situ Pinning for Slipped Capital Femoral Epiphysis (SCFE): Correlation with Clinical Symptoms and Range of Motion of the Hip Joint*

K. Mladenov*, K. Vedder, A. Protzel, U. von Deimling

Asklepios Kinderklinik, Sankt Augustin, Deutschland

***Question***: It has been suggested that decreased anterior femoral neck off-set may produce "cam" like impingement and cause hip joint pain and arthrosis. However the correlation between radiologic findings and clinical range of motion of the hip joint has been very sparsely discussed in the literature.
**Purpose**: To study the clinical correlation between radiologically decreased anterior femoral neck off-set and clinical findings with focus on hip pain and range of motion.
***Methodology***: Retrospective single center study.
The inclusion criteria were: patients with mild to moderate SCFE, treated with "in situ" pinning and followed up for a minimum of 2 years after surgery. The anterior femoral neck off set was evaluated on standardized lateral hip X-rays by means of the "alpha" angle as described by Nötzli. Values of over 50° were considered as pathological. Clinical data for hip complaints, function and range of motion were collected from the patient charts. The Harris Hip score was calculated for every patient. Hips having normal anterior off set (Group I) were compared with the unaffected side and with those having decreased off set (Group II). Focus was placed on Hip flexion, internal rotation and patients complaints.
***Result*** Forty six patients met the inclusion criteria, (22 Group I, 24—Group II). Harris hip score was identical in both groups (99.8 vs. 98.7 points). Hip flexion of affected hips with decreased anterior off set, of affected hips with normal anterior off set and of the unaffected hips was also identical (127.6° vs. 129.7° vs. 130.6°, respectively). Internal rotation of affected hips with decreased anterior neck off set was significantly lower compared to affected hips with normal anterior off set and to unaffected hips (23.3° vs. 36.9° vs. 37°, respectively) and this was independent from the degree oft he initial slip.
***Conclusion***: Decreased anterior femoral neck off set leads to limited internal rotation of the hip joint. On the short term there are no negative effects on hip function and complaints. The correlation between decreased anterior neck off set, decreased internal rotation, femuro-acetabular impingement and hip osteoarthrosis should be studied on the long term, since it make take many years for symptoms to develop.

***Hip dysplasia V2.5***

### *Results of Percutaneous Musculotendinous Release in Children with Hip Dysplasia Secondary to Cerebral Palsy Aged Under 6 Years*

V. Gattung*, P. Bernius, M. Poschmann

Schön Klinikum München Harlaching, Zentrum für Kinder- und Neuroorthopädie, München, Deutschland

***Question*** Beside other factors elevated muscle tonus can lead to hip dysplasia with subsequent luxation in cerebral palsy. Especially hip adducting, hip flexing and medial knee bending muscles play a main role in developing and maintaining a hip dislocation. Due to the maturation potential of the hip, in children younger than 6 years conservative methods like botulinum toxin therapy, physiotherapy or special orthoses may improve centering of the hip. But hips with a Reimers migration index (RMI) more than 40 % require surgical treatment.
In children with cerebral palsy we prefer percutaneous muscle release because we consider it a minimal invasive method with low risks and good results. Therefore we studied the influence of this method on the RMI.
***Methodology*** We retrospectively examined 43 patients suffering from cerebral palsy from 2 to 6 years (mean 4.04 years) and 55 hips. The severity of cerebral palsy was classified according to gross motor function classification system (GMFCS). Patient's GMFCS ranged from 3 to 5. Inclusion criteria were children aged under 6 years with an RMI more than 25 %. RMI was measured in X-rays pre- and postoperatively in a follow up of 11.9 months (0–36 months). Depending on clinical examination of the patients being under anaesthetic immediately before operation, we performed percutaneous release of hip adducting, superficial hip flexing or medial knee bending muscles or combinations of these.
***Result*** Altogether RMI could be improved from 42.5 to 37.8 %.
Concerning hips with a RMI from 25 to 39 % (group A, n = 22) RMI could be improved only slightly (from 30.8 to 29.8 %), whereas hips with a RMI over 40 % (group B, n = 33) RMI could be improved from 50.2 to 43.1 %.
In group A the RMI worsened in 5 hips (22.7 %), stayed equal in 11 hips (50 %) and improved in 6 hips (27.3 %).
In group B the RMI worsened in 4 hips (12.1 %), stayed equal in 12 hips (36.4 %) and improved in 17 hips (51.5 %).
In GMFCS 3 patients, RMI could be improved from 37.1 to 33.6 %, in GMFCS 4 patients from 42.1 to 33.8 % and in GMFCS 5 patients from 46.9 to 45.8 %.

***Conclusion*** Soft tissue balancing is an efficient possibility to avoid further hip migration in children with cerebral palsy aged under 6 years. Although operative hip reconstruction might be necessary in future, early percutaneous muscle release can reach a better situation for further reconstructive surgery.

***Hip dysplasia V2.6***

### *Subtrochanteric Femur Fracture as Serious Complication After Operative Stabilization of Slipped Capital Femoral Epiphysis: A Reason Not to Operate the Non-Affected Hip?*

H. Lengnick*, B. Schultz, P. Gerhard, R. Wegener, H. Klima, E. Payne

Ostschweizer Kinderspital, Abteilung Kinderchirurgie, St. Gallen, Schweiz

***Question*** Slipped capital femoral epiphysis (SCFE) is a common problem in adolescents making operative stabilization most often necessary. Slipping of the contralateral (cl) side is described in 20–60 %, while operative treatment is unclear and controversially discussed 1, 2.
Reported complications after operative treatment in SCFE are chondrolysis, femur head necrosis, premature closure of the epiphysis, infection, implant failure, but not subtrochanteric femur fracture 3.
We present the data of a patient cohort to show complication risk after operative treatment in SCFE and discuss necessity of operative stabilization of the contralateral side.
***Methodology*** Operative treatment of SCFE was performed in 22 hips of 17 patients (male n = 10, female n = 7) by four different orthopaedic surgeons. On average patients were 12.7 (±1.6) years old, 14 showed SCFE acuta, four chronic and four acute on chronic SCFE. Prophylactic stabilization of the contralateral side was done in 12 hip joints. Implants for osteosynthesis after closed reduction (slipping <40°) were Hansson-Pins (SCFE n = 7, cl n = 6), K-wires (SCFE n = 10, cl n = 4) and single screw (SCFE n = 5, cl n = 2). Open reduction (>40°) was necessary in two patients.
***Result*** Overall nine complications (23 %) have been shown: subtrochanteric fractures after adequate trauma in five (13 %), K-wire overlength in three (8 %) and loosening in one patient (3 %). Within the group with subtrochanteric fractures three patients (60 %) showed fracturing of the contralateral side. Concerning subtrochanteric femur fractures Hansson Pins showed the highest rate of fractures in three patients (60 %). Fracture risk was highest within the first 2 months postoperatively.
***Conclusion*** Subtrochanteric femur fracture is a serious complication after operative stabilization of SCFE. In our study fracture risk after stabilization of the contralateral side is comparable with the SCFE side. Possible reasons for this are inadequate operation technique with impairment of the lateral corticales by multiple drilling, patient's constitution and lacking of compliance.
Due to fracture risk for the proximal femur after contralateral stabilization we recommend to carefully evaluate the indication for operation of the contralateral hip taking pain, patient compliance, surgeons' experience and available implant into account.

***References***
[1] Jerre R et al (1994) JBJS 76:563–7
[2] Koczewski P (2001) Chir Ortop Pol 66:357–64
[3] Hefti F (2006) Springer Verlag, 2.Auflage, S.219–26

***Hip dysplasia V2.8***

### *Hip Morphology in MPS 1-H Patients After Hematopoietic Stem Cell Transplantation*

S. Breyer*[1], N. Muschol[2], M. Schmidt[2], K. Babin[1], M. Rupprecht[1], K. Ridderbusch[1], R. Stücker[1]

[1]Altonaer Kinderkrankenhaus, Kinderorthopädie, Hamburg, Deutschland, [2]UKE, Pädiatrie, Hamburg, Deutschland

***Question*** The purpose of this study was to determine the morphology of the acetabulum and the head of the femur in MPS-1H patients using magnetic resonance imaging (MRI) and plain radiographs of the pelvis. Information should help in the preoperative decision making process.
***Methodology*** A retrospective review of patients with MPS-1H was performed. 32 hips were analyzed. Radiographic evaluation of the pelvis was performed on all patients, MRI in eight cases. Hip dysplasia was quantified by acetabular index (AI) as described by Tönnis (Tönnis 1976). As an index of hip stability, the migration percentage of Reimers (MP) was used. (Reimers 1980). The measurements include AI for the labrum, cartilaginous and bony landmarks and MP for the bony and soft tissue coverage. Bone AI on MRI was compared with the same angles on the radiographs.
***Result*** The average age at the time of radiography was 5.0 years. The mean AI in radiography was 36.1°. The mean migration percentage in the radiographic view was 60.6 %. In the MRI group the average age was 6.4 years. The mean radiological AI was confirmed in MRI measurements. The average C-AI was 20.6°. It decreased even more in consideration of the labrum. No difference was seen in the migration percentage of Reimers. As a sign of instability the MP of Reimers in plain radiography, was increased in all hips. The soft tissue coverage was measured in the MP and showed a decreased level of instability. The mean MP corresponding to the cartilage coverage was 45.3 %. In consideration of the labrum, the MP decreased down to levels of 27.5 %.
***Conclusion*** Our results establish that patients with MPS-1H have a three times greater cartilaginous coverage then healthy kids. Only radiological criteria can leads to misunderstanding the morphology. MRI measurements can document the cartilaginous coverage and help in decision making processes. Our findings suggest that MRI promotes more accurate selection of children for pelvic reconstruction. Further multicentric studies have to confirm this.

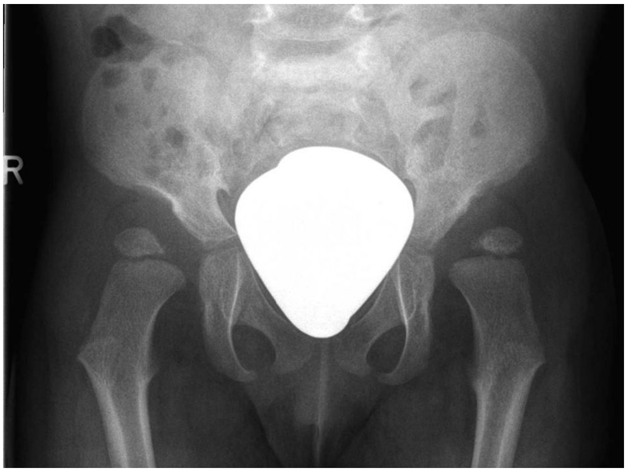

Hipmorphology

***Hip dysplasia V2.9***

### *Gait Deviations After Perthes Disease in Dependence of the Radiological Outcome*

B. Westhoff*, A. Müller-Reinartz, L. Hegemann, C. Zilkens, D. Rosenthal, R. Krauspe

University of Duesseldorf, Medical Faculty Department of Orthopaedics, Duesseldorf, Deutschland

***Question*** Depending on the final extent of hip deformity Perthes disease will lead to early osteoarthritis. The aim was to analyze the functional outcome and to correlate the results with the radiological outcome.
***Methodology*** 30 adults (mean age 31 + 11 years) treated for unilateral LCPD were investigated clinically, radiographically and by 3D-gait analysis. The clinical result was rated by the Harris Hip Score (HHS). According to the Stulberg-rating system patients were divided in 2 subgroups: group 1 = spherical joint (Stulberg type 1 and 2, n = 16), group 2 = aspherical joint (Stulberg type 3–5, n = 14). 3D-gait-analysis was performed with a VICON 512 system. Patients walked at a self-selected speed–barefoot. Spatiotemporal, kinematic and kinetic parameters were evaluated and compared to a group of normal adults (n = 40, average age 28.0 years). Statistical analysis was performed by the Mann–Whitney-U test for independent samples; the correlation within in the patients group was performed using an ANOVA-analysis and the Kruskal–Wallis-test for non-parametric samples; a p value of less than 0.05 was considered to indicate statistical significance.
***Result*** Patients with a spherical joint showed a significantly better clinical results (HHS 89 vs. 78 p., p = 0.019) and less degenerative changes. Gait deviations were more severe in group 2: asymmetry related to the duration of stance phase was more pronounced; in sagittal plane kinematics ROM of the hip and knee joint were reduced, asymmetry between involved and non-involved side was more pronounced too; in frontal plane ROM of the pelvis and trunk was reduced as well; power generation at the hip joint was impaired as well.
***Conclusion*** Gait analysis after LCPD showed significant deviations of the gait pattern in comparison to the controls with loss of symmetry. These are part of an unloading mechanism. The deviations are more pronounced in case of a poor radiological outcome. Further studies are necessary to determine functional predictors for the development of secondary osteoarthritis which may than be influenced by conservative or surgical treatment options.
Compared to early follow-up examinations after LCPD (Westhoff, Int Orthop 2011) gait deviations are more pronounced in early adulthood.

## Best Lectures

***Best lectures V-Top***

### *Treatment of Congenital Wry–Neck (Torticollis) by Dreiss' Method and Computer Aided Analysis of the Face Asymmetry*

L. Schwering*[1], V. T. Förster[2]

[1]Mathias-Spital, Technische Orthopädie, Rheine, Deutschland, [2]Universitätsklinikum Freiburg, Department Orthopädie Traumatologie, Freiburg, Deutschland

***Question*** The treatment of wry neck changed from the first operative intervention by Isaac Minnius 1642 to modern pediatric orthopedic concepts. In allusion to Foederl's approach G. Dreiss developed a concept for the treatment of this deformity in the early seventies of the last century. The purpose of this investigation is to demonstrate the efficiency of the procedure and the control of the scoliosis of the skull by graphic programs (Corel Draw 12 and Adobe Photoshop CS4).
***Methodology*** In the pediatric orthopedic practice wry neck is primary treated by conservative means like physiotherapy. The success rate is reported to be 90 % and more (H. Binder 1987). During the last 10 years 14 children (9 boys and 5 girls) had to be treated operatively finally. The mean age at operation was 6 years and 4 months. The Dreiss' procedure consists from triterminal tenotomy of the sternocleidomastoid muscle in a special way. Postoperative a removable diadem thermocast was applied for four to 6 weeks depending on the patientś age and the severity of the deformity. A digital picture of the patientś face was documented pre- and post-operatively. After dividing the face in the midline two new "faces" from two left and two right parts were created. Using this graphic controlled method the improvement of the asymmetry of the face was detected.
***Result*** The mean follow up was 7 years and 10 months. During the operation no complication (especially damage to the facial nerve) was observed. Range of movement to the restricted side improved but could not reach the same range of motion as on the other side in children older than 6 years at the intervention. The results of computer aided analysis illustrated a harmonization between the two artificial faces.
***Conclusion*** The Dreiss' procedure in the treatment of wry neck proved to be an excellent method compared with the existing literature. With the number of patients it might be useful to operate children with wry neck under the age of six to reach a complete cure. The computer aided analysis is a noninvasive tool that helps to show the improvement of facial asymmetry.

***Best lectures V-Top***

### *Criteria for Successful Arm-Prosthetic Supply in Childhood and Adolescence*

M. Horter*[1], J. Thormann[2], G. Gosheger[3], H. Kirch[2], R. Rödl[1], H. H. Wetz[2]

[1]Kinderorthopädie, Deformitätenrekonstruktion und Fußchirurgie, Universitätsklinikum, Münster, Deutschland, [2]Klinik für Technische Orthopädie und Rehabilitation, Universitätsklinikum, Münster, Deutschland, [3]Klinik für Allgemeine Orthopädie und Tumororthopädie, Universitätsklinikum, Münster, Deutschland

***Question*** Which criteria have to be considered to achieve a successful arm-prosthetic supply? What do acceptance and compliance of prosthetic devices in childhood and adolescence depend on? Does family influence the children's attitude towards disability and acceptance of the prosthesis and how could this effect be used for therapy in a positive way?
Do children even need prosthetic devices?
***Methodology*** We examined 51 patients (0–16 years at first contact, 21 girls, 30 boys) based on a retrospective data analysis and a self-made questionnaire for children and their parents. Defined criteria were: sex, diagnosis, kind of prescription, age of child at the date of first contact with the clinic and of first prosthetic supply, wearing behaviour and wearing time in hours per day, reasons for rejection, stigmatisation of parents and their children, selective wearing of the prosthesis, the effect of group behaviour on other children, and proposals for improvement.
***Result*** The patient's sex shows influence of the arm-prosthetic acceptance: 75 % of the female patients use their prostheses, but only 33 % of the male patients. Amputated patients wear their prostheses more often than patients with congenital deformities. The average age at the date of first prosthetic supply was 4.6 years. The success rate decreases mainly at the male patients with increasing age of first prosthetic supply. Besides the type of prescription, stigmatisation of the parents as well as the child's environment shows a latent impact on compliance and consequent acceptance of the prosthetic device. The results of this study are congruent with current literature.
***Conclusion*** Orthopaedic specialists, parents and the children should decide together about best time and need of prosthetic supplies. This study reveals the difficulty in finding Methodology criteria, which promote the acceptance. The environment and the education of the child show major effects. It is not possible to generalize if the supply of a child with a prosthetic device is absolutely necessary, but the child should have the opportunity to decide on its own. Current literature especially emphasizes the amenities of prostheses concerning the physical and psychological development. Apparently the practical experience differs: Half of the children do not even wear the prosthetic device.
Even tests published in preceding studies that intend to set up a classification system for the prosthesis wearing behaviour do not legitimise the rejection to supply a child with prosthetic device.

***Best lectures V-Top***

#### *Experiences with Clubfoot Therapy in Older Children in Germany*

U. Brückner*[1], A. Reeg[2], B. Schnuck[1], M. Schulte[1]

[1]Agaplesion Diakonieklinikum Rotenburg, Kinderorthopädie, Rotenburg/Wümme, Deutschland, [2]Stiftung Feuerkinder, Berlin, Deutschland

***Question*** In 2012 the Deutsche Gesellschaft für Orthopädie und Orthopädische Chirurgie (DGOOC) introduced revised guidelines for the treatment of clubfoot (cf). These guidelines include the Ponseti therapy as a standard for newborns but also focuses on the treatments of relapses in children up to the age of 3 years.
However, in German-speaking countries there exist no guidelines for the therapy of untreated clubfoot cases and relapses for older children (>3 years of age). As a result, clubfoot therapy is applied on a case-by-case basis.
***Methodology*** The aim of this paper is to identify and adopt a national guideline for the therapy of clubfeet in children older than 3 years of age. In order to gain insights on the current practices, standards as well as the short- and long-term treatment results of clubfoot therapy in Germany we carried out expert interviews in several orthopaedic departments specialised on children.
***Result*** The best way of treatment seems to be the initial Ponseti therapy combined with minimal surgery, depending from the result of the casting.
***Conclusion*** In line with international experiences our data suggests that the standard Ponseti therapy offers the most promising treatment results in cases of older children. We therefore recommend the DGOOC guidelines to be extended to children older than 3 years.

***Best lectures V-Top***

#### *A Critical View on In-Patient Treated Hip Luxation in the Era of Ultrasound Screening*

A. Postler*, K.-P. Günther, F. Thielemann

UniversitätsCentrum für Orthopädie und Unfallchirurgie, Dresden, Deutschland

***Question*** In spite of the dutifully performing ultrasound screening in Germany there have been repeated cases of dislocated hips, which aren't be detected, even treated on time. The aim of the current study was therefore to investigate the courses of those children, who need an in-patient treatment.
***Methodology*** In this retrospective cohort study 74 children (62 % female, 12 % male) with hip dislocation, who needed an in-patient treatment between January 2001 and February 2014 were included. Children with neuropathic, myopathic or teratological luxation of the hip were excluded.
Main risk factors, date of first ultrasound screening, kind of pre-treatment and the subsequent procedures were recorded.
***Result*** Four children got their first ultrasound screening delayed after the 6th week of life and six children had no screening at all.
54 Children (73.0 %) were advised after the 6th week of life. 43 patients had a previous treatment for the hip dislocation, three in foreign countries. Prior treatment involves therapy by various bandages (41.9 %), overhead-extension (5.4 %), spica cast (1.4 %), multiple forms of noninvasive therapy (12.2 %) or even frustrating surgical procedures (4.1 %).
In 60.8 % (n = 45) an initial closed reduction and spica cast immobilization for 12 weeks was successful. Five of those children (6.8 %) needed a two-step closed reduction. In 39.2 % (n = 29) of the children an open reduction and postoperative immobilization was performed.
Because of persistent dysplasia of the acetabular roof and instability 24 children (12 patients from the closed reduction group, 12 patients from the open reduction group) needed further operative procedures. Those secondary operative procedures were usually performed after a period of prolonged conservative treatment at an average age of 27.6 months (range 10–74 months).
***Conclusion*** The results of the current study emphasize the importance of the early ultrasound screening and adequate therapy in cases of a positive family history and breech position. Delayed detection of the hip dislocation without any prior treatment (32.4 %, n = 24) was the main factor for open reduction (75.9 % of all open reduction cases), respectively secondary surgical procedures to stabilize the hip.
It should be assumed that a frustrating previous treatment will be a further reason for any necessary in-patient management. An experienced out- and in-patient treatment is therefore mandatory.

### Axial Deviations/Limb Lengthening

***Axial deviations/limb lengthening V4.2***

#### *Principle, Indication and Midterm Results of the Femoral Intertrochanteric Valgus Osteotomy in Legg–Calvé–Perthes Disease*

B. Heimkes*, B. Weiß, C. M. Ziegler, S. Utzschneider

Klinikum der Universität (LMU), Campus Großhadern, Klinik für Orthopädie, Physikalische Medizin und Rehabilitation, München, Deutschland

***Question*** Occurrence of hinge abduction is a negative prognostic factor in Legg–Calvé–Perthes disease. For this problem Catterall proposed femoral valgus (extension) osteotomy as a salvage procedure.
Purpose of the retrospective study was to evaluate the indications and results of this procedure in our patients with Legg–Calvé–Perthes disease.
***Methodology*** 28 patients who underwent the procedure between 2004 and 2013 were examined clinically and radiologically at an average of 5.5 years (range 1.0–10.5 years) after surgery.
***Result*** Indication. The mean age at surgery was 10.7 years (range 6.2–16.3 years). 96.4 % of the patients were pretreated conservatively, 10.7 % additionally by pelvic osteotomy, 25.0 % by varus femoral osteotomy and 3.6 % by tissue release. Staging at time of procedure was: 10.7 % condensation, 50.0 % fragmentation, 14.3 % reossification and 25.0 % remodelling stage. Grading: 0 % were classified Herring A, 50.0 % Herring B and 25 % Herring C. 25.0 % could not be classified. Follow up: (1) The hip abduction increased 30.0° (range −2.5° to 75.0°) from preoperative 15.0° (range −30.0° to 50.0°) to postoperative 40.0° (range 15.0° to 65.0°). (2) The average neck-shaft angle changed from preoperative 129.5° (range 107.8°–149.6°) to postoperative 149.0° (range 131.6°–168.1°). (3) Final radiographic outcome according to Stulberg: Type II deformity 7.1 %, Type III 39.3 %, Type IV 21.4 %, Type V 7.1 %. 25.0 % of the patients could not be classified yet.
***Conclusion*** According to the results femoral valgisation is recommended both as a salvage procedure after failed previous osteotomies as well as a first procedure for hips with hinge abduction.

***Axial deviations/limb lengthening V4.3***

### *Guided Growth of the Infant's Skull: A Pediatric Orthopedic Method?*

H. Willenborg*, S. Martin

Orthopädie der MHH im Annastift, Kinderorthopädie, Hannover, Deutschland

***Question*** Guided growth of the infant́s skull—a pediatric orthopedic method?
How long should we observe the development of an asymmetrical development of the infant́s skull? When should we intervene by guiding growth? In which cases is the conservative approach not indicated? When should we proceed to surgery?
***Methodology*** Infants are impeded in their healthy development by asymmetry. This deformity may lead to life-long problems.
Asymmetry in infants is often multifactorial and can involve the entire body.
Individual aspects can aggravate each other: asymmetrical positioning of the feet, impeded hip abduction (with or without DDH), asymmetrical hip rotation, reversible blockages of the pelvis, the sacroiliac joint or the upper cervical spine with or without wry neck etc.
If the infant is denied the variation of positioning during the first months of life and is constantly kept on its back (not only during sleep as advised by the German association for sleep medicine) a deformation of the soft skull may result. Non-orthotic treatment can be of help up to the age of 6 months. Because of the hardening of the skull there is no realistic chance of achieving symmetry "spontaneously" beyond this age.
Depending on the severity of the skull deformity, guided growth through helmet therapy can be initiated as early as an age of 4 months (or later) to achieve symmetry for the skull, the face and the axis of the ears.
***Result*** Different methods are needed for the treatment of the vicious circle of "asymmetry syndrome" (positioning, chiropractic, physiotherapy, bandaging, casting etc.). Pediatric orthopedists should be enabled to perform a holistic assessment and examination of the infant. Helmet therapy does not only lead to a symmetrical shape of the skull but often also contributes to a resolution of the more complex "asymmetry syndrome".
On principle a cranio synostosis cannot be treated by helmet therapy. In severe cases this malformation is an indication for surgery. If synostosis and positioning problems occur together, both: conservative and surgical approaches, can be combined.
***Conclusion*** Helmet therapy is a very valuable contribution to pediatric orthopedic treatment.

***Axial deviations/limb lengthening V4.4***

### *Correction of Static Axial Alignment in Children with Knee Varus or Valgus Deformities Through Growth Guidance: Does It Also Correct Dynamic Frontal Plane Moments During Walking?*

H. Böhm*[1], F. Stief[2], K. Sander[3], C. Multerer[1], L. Döderlein[1], M. Hösl[1]

[1]Orthopädische Kinderklinik, Kinderorthopädie, Aschau, Deutschland, [2]Orthopaedic University Hospital Friedrichsheim gGmbH, Movement Analysis Lab, Frankfurt/Main, Deutschland, [3]Universität Jena, Waldkrankenhaus GmbH, Eisenberg, Deutschland

***Question*** Malaligned knees are predisposed to the development and progression of unicompartmental problems because of the excessive load placed on one side of the knee. Therefore guided growth in skeletally immature patients is recommended. Indication for correction of varus/valgus deformities are based on static radiographs. However the internal knee abduction moment, a valid marker of mechanical wear at the knee joint during walking, showed only weak correlation to malalignment determined by static radiographs. So far, none of the studies about guided growth reported the effect on the loading situation during walking. This is astonishing since the procedure aim to prevent excessive load placed on one side of the knee. Therefore, the aim of the study was to measure the effects of growth guidance on the normalization of frontal plane knee joint moments during walking. The hypothesis was that the change in dynamic moments and the change in static alignment are closely related.
***Methodology*** 8 patients (11–15 years) with idiopathic axial varus or valgus malalignment participated. 16 typically developed peers served as controls. Gait analysis and clinical assessment were performed the day before implantation and explantation of eight plates. The static mechanical tibiofemoral axis angle (MAA) was calculated from a captured standing trials and radiographs. The dynamic frontal plane knee moments were calculated as the average over the stance phase of gait. A total of 15 legs was individually analyzed pre and postoperatively with respect to controls. Correlation between static MAA and dynamic frontal plane knee joint moments and their change by guiding growth were performed.
***Result*** The changes in dynamic knee moment in the frontal plane following guiding growth showed excellent and significant correlation to the changes in static MAA ($R = 0.97$, $p < 0.001$), so that the hypothesis of the study turned out to be correct. Contrary to the correlation of the changes, there was no correlation between static and dynamic measures in both sessions. This can be negative when a natural loading situation before treatment turned into a pathological one after treatment.

***Conclusion*** In conclusion guiding growth has a predictable effect on the dynamic load situation during walking. Gait analysis might be useful to assess the preoperative load distribution during walking; it might reveal individual gait pathologies that might further affect dynamic joint moments in cases where the static situation has been already corrected.

***Axial deviations/limb lengthening V4.5***

### *Guiding Growth Supports Correction of Equinus in Residual Paediatric Clubfoot*

V. T. Förster*[1], L. Schwering[2]

[1]Klinik für Orthopädie und Unfallchirurgie, Universitätsklinikum, Freiburg, Deutschland, [2]Mathias-Spital Rheine, Technische Orthopädie, Rheine, Deutschland

***Question*** After clubfoot correction pathologic ankle joint angles may remain and result in unphysiologic gait with extended forefoot load. Neither a new release nor tendon lengthening can solve this problem. The purpose of this clinical trial is the answer to the question if guiding growth can be useful in the correction of this pathology of the tibial pilon.
***Methodology*** Eight boys and two girls with 16 feet in equinus position because of pathologic joint surface angles of the distal tibia between 95° and 100° were treated by the means of guiding growth. Five boys presented seven previous treated idiopathic clubfeet, one boy after achilles tendon lengthening (ATL) due to muscular dystrophy in both feet and one girl also after ATL because of congenital arthrogryposis multiplex. To improve gait and make the wear of splints easier one 8-plate was applied strictly in ventral position of the distal tibial growth plate. Patientś files and radiographs were evaluated.
***Result*** During a mean follow up of 2 years an 17 months and a mean duration of the 8-plate in situ of 20 months a relevant improvement of the distal tibial joint surface angle near 80° (Paley) was observed. In one boy the correction was incomplete in two feet because of maturity ahead of time. In one boy’s foot a moderate overcorrection was achieved voluntarily. Neither during the implantation of the 8-plate nor during the hardware removal any complication was detected. The mean operation time from incision to suture lasts 33 min/8 plate.
***Conclusion*** Guiding growth seems to be a useful tool in the correction of enhanced distal tibial joint surface angles in children. Because of the minimal invasive approach and immediate full weight bearing postoperative this seems to be suitable for children. This intervention can restore foot function by normalization of tibial pilon. Meticulous preparation and exact application of the 8-plate are prerequisites for a trial free from complications.

***Axial deviations/limb lengthening V4.6***

### *Lengthening and Correction of Axial Deviations in Children*

M. Schmidt*, G. Salameh

Waldhofzentrum, Kinderorthopädie, Kronberg, Deutschland

***Question*** Limb lengthening and deformity correction in children has in most cases obstacles and complications during treatment like axial deviations s, knee flexion contracture, drop foot and deformity in addition to congenital and acquired malalignment. This need a special method and strategy for successful treatment
***Methodology*** The external fixation hinge system (Salahmehfix) is an arch hinge system with various diameters to assemble the shape of the extremity with special connecting hinges to correct axial and rotational deviations.
The stability of the simple functional arc system is high, it has a good tolerance by the young patients.
It can be modified during the treatment if needed to achieve full restoration of the extremity.
***Result*** From 1995 to 2013 this system was used in 548 children with different indications in the lower and upper limbs. They presented with axial deviations, limb length discrepancies and combined deformities.
Results were excellent in 312 cases, good in 164 cases, fair in 84 cases and 6 had poor outcome.
Complications were mainly superficial pin infection, pin breakage, but no nerve or vascular complication was seen.
***Conclusion*** The new developed external fixation system allows the correction of Axial deviations, length discrepancy, contractures and even combined complex deformities.
The new system provide high stability, high tolerance by the children, fast weight bearing and easy handling by the surgeon.
The treatment is not easy and experience is essential to get a successful result.

***Axial deviations/limb lengthening V4.7***

### *Limb Lengthening by External Fixation Techniques in 41 Patients Affected by Proximal Femoral Focal Deficiency and Fibular Hemimelia*

H. Bonnyai*, M. Klein, S. Nader

Schön Klinik, Klinik für Kinderorthopädie, Vogtareuth, Deutschland

***Question*** The limb lengthening technique introduced by Gavril Ilizarov and further developed over the past three decades has increasingly become an alternative to amputation for patients affected by Proximal Femoral Focal Deficiency (PFFD) and/or Fibular Hemimelia (FH). It is challenging, causes complications, but may be termed successful after all.
The aim of this study was to examine the lengthening distance and the lengthening index of the limb lengthening technique of the lower extremity in our department, as well as to analyze its complications.
***Methodology*** 41 patients with PFFD and/or FH who had all together 43 limb lengthening procedures with an external fixator, either a Taylor Spatial Frame or a Mono Rail System, were studied. Surgery reports, discharge letters and X-rays of the patients dating from between 2008 and 2013 were evaluated and examined, including major and minor complications, as well as the therapy thereof.
***Result*** The average lengthening distance was 87 mm (Femur: 74 mm, Tibia: 93 mm) over an average period of 366 days. Thus, the average rate of lengthening was 0.27 mm/days. In other words, the average healing index was 3.76 days/mm. Major and/or minor complications were observed during almost all lengthening procedures. Complications included a pin or a wire failure, loosening or local infection, traumatic fractures of the bone to be lengthened, reduction in range of motion in the knee, premature bone healing, and subluxation. Most complications were temporarily and could easily be treated. Therapy was either conservative or operative, depending on the extent of the complication.
***Conclusion*** Limb lengthening is a viable therapy in children with PFFD and/or FH. Patients and parents have to be aware of the complications though; complications are common but manageable and mostly preliminary. Regular physiotherapy and orthesis are essential for the success of limb lengthening with the external fixator.

***Axial deviations/limb lengthening V4.9***

#### *The Correction in Complex Deformities of the Femur as in Proximal Focal Femur Deficiency (PFFD) Performing Double Osteotomy of the Femur and Stabilization with an External Fixator*

H. Forkl*, S. Nader

Schön Klinik, Klinik für Kinderorthopädie, Vogtareuth, Deutschland

***Question*** PFFD is a rare congenital deformity of the femur associated with leg length discrepancy, deformity of axis and abnormity of femoral torsion. After analyzing the axis of the limb double osteotomy of the femur is often required to correct the deformities. The correction is aimed on the predefined position to achieve physiological axis and torsion and the same length of legs.
***Methodology*** This study includes 6 patients with PFFD requiring double osteotomy of the femur and stabilization with an external fixator. All cases showed a multilevel deformity of the femur that had to be corrected.
Preoperative radiological diagnostic like X-ray of the femur (a.p. and lateral), MRI measurement of the torsion and X-ray of the hand to determine the skeletal age were done followed by digital analysis of the deformity and definition of the CORAs. The present discrepancy of the length and the expected discrepancy at maturity were calculated by the multiplier method. The complexity of the deformities required double osteotomy of the femur and application of an external fixator. The osteotomy was performed following the plan and the external fixator was attached to the bone on predefined position. The achieved correction of the axis, the distance of lengthening and complications were recorded.
***Result*** In all patients double osteotomy of the femur was performed. The age of patients was 9.5 (±3.9) years. The radiological outcome showed in a.p. view an average deformity of 28° proximal and 18° distal. In the lateral view it was 16° (±9). The AT of the femoral neck was reduced by 24° compared to the other side. The proximal deformity in a.p. view could be corrected to an NSA of 124° (±12.3). The mLDFA could be corrected to 87° (±1.2). The mean lengthening resulted in 50 mm. The fixator was removed after 277 days in average. There were no intraoperative complications. During lengthening process infections of the pins and reduction of the ROM of the knee joint were recognized.
***Conclusion*** Double osteotomy of the femur and use of an external fixator to correct deformities in cases of PFFD represents a save method of correction with the option for limb lengthening. Accurate preoperative planning is important. By using this method the deformities can be corrected on different levels simultaneous causing only low soft tissue trauma. Postoperative the axis and torsion can be adjusted during the limb lengthening to achieve the best result. The rate of complications intraoperative and postoperative is low.

### Disturbed Growth of Spine/Deformities of the Feet

***Disturbed growth of spine/deformities of the feet V5.1***

#### *3-D-Gait Analysis and 4-D-Body-Surface Changes in the Effect of Hypercorrection Braces ("Mirror-Braces") in Left Lumbar Scoliosis (LENKE Type V/VI AIS)*

J. Matussek*, E. Dingeldey, J. Wolfsteiner

Orthopädische Klinik Universität Regensburg, Kinderorthopädie, Regensburg, Deutschland

***Question*** To avoid overblown expectations of patients and their families in the conservative AIS treatment, a realistic prognosis of the proposed treatment path has to be defined after about 4 months of in-brace treatment and to be followed up over the whole treatment period. X-Ray alone is not capable to describe the postural problems in stance and gait that occur in left lumbar scoliosis (LENKE V/VI or King I) which is mechanically very different from typical thoracic curvatures in AIS. Active correction of the childs postural asymmetry of the spino-pelvic-complex -as it has to be addressed to during brace treatment and learned in physiotherapy programmes- is a great challenge for the individual and the medical and orthotists team. 3-D-Gait data, 4-D surface.
***Methodology*** 30 female IS patients with defined lumbar curvatures: Lenke 5C/6C were treated with braces of a straightforward hypercorrection design

- Risser 0–2
- Bracing for an average of 16–21 h a day
- 3 D-gait analysis at initial contact and after 12 months and surface stereometric measurements with the 4D-formetric system were performed initially and at every clinical control date.
- X-ray control at 4(6)/12/(24) months in the brace in two planes.
- Initial Cobb angles ranged between 20° and 45° lumbally.
- Typical physiotherapy programme (Schroth) (2×/week).
- 3 D gait data of the spino-pelvic complex were obtained.

***Result*** Once the primary 3 D-correction after 4 months of brace treatment and physiotherapy programme valued more than 40 % (>1/3) (n:30) King I/II curvatures), these improvements could be maintained or even improved during the next 20 months. Hypercorrective braces yielded a significantly better primary Cobb angle improvement and a better surface scan- and 3-D-gait symmetry.
The active correction without the brace, documented with the 4D-Formetric-System did not immediately correspond with changes of the Cobb angle, though usually after a delay of 6 months of repeated training, active improvements could be documented in 30 patients.
In 7 patients, stereometric measurements were not immediately conclusive. With a primary correction <40 % (n:7)in the brace, we observed a further loss of achived improvements of the Cobb angle at start towards the end of skeletal growth. 3 D Gait data showed significant changes of the spinopelvic complex after 12 months of effective treatment.
***Conclusion*** The typical gait and optometric changes of LENKE V/VI curves have to be sufficiently addressed in the proposed brace designs.

***Disturbed growth of spine/deformities of the feet V5.2***

#### *Rib to Pelvis VEPTR Construct Treatment Duplicates Normal Height Gain of Lumbar Vertebral Growth in Neuromuscular Scoliosis*

N. Flöter*[1], C. Hagemann[2], K. Ridderbusch[1], M. Rupprecht[1], R. Stücker[1], P. Kunkel[2]

[1]Altonaer Kinderkrankenhaus, Kinderorthopädie, Hamburg, Deutschland, [2]Altonaer Kinderkrankenhaus, Pädiatrische Neurochirurgie, Hamburg, Deutschland

***Question*** Neuromuscular scoliosis frequently emerges at a young age and fast progression rate. In non-ambulatory patients vertical expandable prosthetic titanium rib (VEPTR) constructs attached bilaterally between upper thoracic ribs and iliac crest are growth preserving and thus often the treatment of choice.
In this study we want to evaluate the impact on growth patterns of vertebrae within the length of distraction exerted by this devices.
***Methodology*** Twenty-six children (16 male, 10 female; mean age at index surgery 8.6 years) were treated between 2008 and 2014 with bilateral VEPTR constructs. 6 patients had scoliosis due to spasticity, 7 had myopathy or muscular dystrophy, 5 spinal muscular atrophy and 8 patients syndromic diseases. Patients with spina bifida were excluded because of their altered lumbar vertebral anatomy. Vertebral height and width of the topmost thoracic vertebra within instrumentation level (usually T3) and height and width of L5 were measured in post-index and post-final follow-up X-rays. The amount and velocity of growth was calculated for each vertebra and compared to normal growth data from the literature.
***Result*** The average follow-up period was 41 month (range 6–78 months). Lengthening was performed regularly every 6 months. The mean height gain per year was 1.9 mm in L5 and 0.5 mm in the thoracic vertebrae. The mean width gain per year was 0.2 mm in L5 and 0.7 mm in the thoracic vertebrae. The difference between height gain per year of L5 and the thoracic vertebrae was highly significant ($p = 0.0002$). No significant difference could be shown between the width gain of lumbar and thoracic vertebrae.
***Conclusion*** Normal height and width gain of each lumbar vertebra is approximately 1 mm per year for children between 5 and 15 years of age. The thoracic vertebrae only increase their size at a rate of about 0.5 mm in height and width.
In this study the growth velocity in height of L5 under vertical traction was twice as much as normal whereas width gain was reduced. This effect could not be shown in the thoracic spine within the traction length. The Hueter–Volkmann principle states that appendicular growth plates kept under traction tend to grow faster because of load reduction. In the thoracic vertebrae stabilized by the rib cage this unloading effect seems to be of less impact.
It can be concluded that the longitudinal growth of the lumbar vertebrae is not only preserved but also stimulated in neuromuscular patients treated with bilateral rip to pelvis VEPTR constructs.

***Disturbed growth of spine/deformities of the feet V5.3***

### *Increased Risk of Pathological Fractures Due to Vitamin D Deficiency in Handicapped Adolescent Patients: Clinical Features, Diagnostic and Substitution*

E. Payne*[1], H. Lengnick[1], H. Klima[1], U. Probst-Scheidegger[2], D. l'Allemand-Jander[2]

[1]Children's Hospital of Eastern Switzerland, Department of Paediatric Orthopaedics, Laboratory for Motion Analysis, St. Gallen, Schweiz, [2]Children's Hospital of Eastern Switzerland, Department of Paediatric Endocrinology and Diabetology, St. Gallen, Schweiz

***Question*** Bone fractures in paediatric orthopaedic patients with chronic diseases and severely handicapped, immobile persons may be due to Vitamin D deficiency. In Switzerland, 20–60 % of the children have decreased Vitamin D levels, so that above all, patients with chronic pain for unknown reason as well as immobile patients e.g. with severe cerebral palsy (CP) GMFCS IV and V are at risk for rickets. Chronic fatigue, progressive muscle pain, weakness and convulsions but also increased infection rates may even be symptoms of Vitamin D deficiency in healthy population.
In order to recognize Vitamin D deficiency in paediatric orthopaedic patients and to avoid further complications, we present a check list for bone health in otherwise healthy children and immobile patients with cerebral palsy.
***Methodology*** In our outpatient paediatric orthopaedic clinic we routinely evaluated a check list filled out by parents, doctors and nurses and control the Ca/25–OH Vit D status as well as Phosphate, Mg, alk. Phos., PTH and Ca/Crea ratio in urine once a year, in all 8–18 year old immobile CP patients. In other patients, symptoms and signs of Vitamin D deficiency are routinely assessed.
***Result*** 36 of 37 CP patients had decreased Vitamin D levels. In one third, pathological bone fractures or pain were found in Vitamin D deficient patients. Handicapped paediatric patients with chronic illnesses or syndroms without trauma presented loss of walking ability due to Vitamin D deficiency.
In healthy adolescents bone pain, e.g. accompanied by MRI controlled bone oedema, was potentially the consequence of hypocalcaemic rickets.
***Conclusion*** Our serial examination could assure that severely handicapped patients with CP regularly present a Vitamin D deficiency. Substitution of Vitamin D should be considered to achieve a release of chronic pain in healthy adolescents. Careful anamnesis as well as Lab control of Ca and phosphate metabolism should consequently and routinely be integrated in our medical treatment concept.
Beside Vitamin D substitution in patients with intestinal-, liver- and kidney diseases all children and adolescents with anticonvulsive medication should retain Vitamin D supply as well as paediatric orthopedic patients with CP level GMFCS IV and V to avoid further serious medical problems.

***Disturbed growth of spine/deformities of the feet V5.4***

### *Radiologic Results and Quality of Life Following Scoliosis Surgery in Patients with Severe Cerebral Palsy*

T. L. Schulte*[1], K. Beckmann[1], G. Gosheger[1], A. Schulze Bövingloh[1], M. Borowski[2], U. Liljenqvist[3], V. Bullmann[4], T. Lange[1]

[1]Klinik für Allgemeine Orthopädie und Tumororthopädie, Universitätsklinikum, Münster, Deutschland, [2]Westfälische Wilhelms-Universität Münster, Institut für Biometrie und Klinische Forschung, Münster, Deutschland, [3]St. Franziskus-Hospital Münster, Wirbelsäulenchirurgie, Münster, Deutschland, [4]St. Franziskus-Hospital Köln, Wirbelsäulenchirurgie, Köln, Deutschland

***Question*** Patients with severe cerebral palsy (CP) often develop neuromuscular scoliosis. Based on the reduced general health status and high surgical morbidity there is often the question of extent of surgical correction and also if patients/caregivers assess surgery as beneficial in terms of quality of life. Aim of the study was to analyse radiological and clinical results after posterior-only and anterior-posterior instrumentation of CP scoliosis.
***Methodology*** In a retrospective cohort study CP patients who underwent posterior or anterior-posterior instrumentation in one institution between 1997 and 2012 with a minimum follow-up of 2 years and a complete set of data were analysed. Patients records and radiographs were studied. Quality of life was assessed using an established questionnaire (Bridwell, Lenke et al., Spine 1999) for neuromuscular scoliosis patients/caregivers considering surgical success, need for care, function, respiratory symptoms, pain, etc. resulting in a total score ranging from $-34$ (worst) to $+34$ (best).
***Result*** 46 patients were included with a mean follow-up of 49 months. 29 patients with posterior-only instrumentation (group P; age 15.1 years; 11 m, 18 f; GMFCS 4.6; BMI 16.7; 12 patients with preoperative respiratory complaints), 17 with anterior-posterior (group AP; age 16.2 years; 9 m, 8 f; GMFCS 4.9; BMI 14.3; 8 patients with respiratory complaints). In group P the Cobb angle of the main curve before surgery was 86°, 40° after surgery, 44° at follow-up, in group AP 107°, 54° and 55°. Preoperative flexibility was the major predictor for relative curve correction. Type of surgery, preoperative pelvic obliquity, C7 plumb line, BMI, GMFCS and gender did not significantly affect relative correction. Complications (defined by Master et al., Spine 2011) occurred in 21 % in group P and 47 % in group AP.
In 96 % of cases surgery was assessed as success. The total score in life quality was +11.7 with no significant difference between both groups. Also results of life quality in detail did not differ. High preoperative Cobb angles correlated significantly with improved quality of life at follow-up. Patients with preoperative respiratory complaints and patients with perioperative complaints did not perform worse than those without.
***Conclusion*** All in all, this cohort presented good radiologic results comparable with the literature. Quality of life at follow-up was good (96 % success). Posterior-only instrumentation showed similar results compared to combined surgery, but with less morbidity.

***Disturbed growth of spine/deformities of the feet V5.5***

### *Ventral Epiphyseodesis of the Distal Tibia for Treatment of Limitation of Passive Dorsalextension*

D. Lewens*, A.-C. Kutschan, C. U. Dussa, L. Doederlein

Orthopädische Kinderklinik, Kinderorthopädie, Aschau, Deutschland

***Question*** The commonest gait disturbance following a successfully treated clubfoot is premature heal raise while walking. This deformity is due to insufficient dorsal extension in the ankle joint. Although several methods have been described aiming to improve the dorsal extension, none of them can be a universal solution to the problem. Of the several methods available, the temporary epiphyseodesis of the distal tibia is a simple technique to change the geometrical configuration of the distal tibia to facilitate the dorsal extension of ankle joint. We present our experience with this technique.
***Methodology*** Since 2011, 6 patients with 9 feet were diagnosed to have a structural equines deformity of the ankle joint leading to restriction of dorsal extension. There age group was between 8 and 15 years. These patients were treated with ventral epiphyseodesis of the distal Tibia. The mean follow-up was 11 months (7–29 months). The degree of correction was measured clinically using the range of motion as well as radiologically using ADTA angle.
***Result*** The passive dorsiflexion improved significantly (ROM >5° DE) in only 2/9 cases. In rest of the cases the passive dorsal extension remained unchanged. There was no diminished plantar flexion. However radiologically, a mean change in the ADTA angle of 6.6°° was noted (2.6°–16.4°). There were no intra or postoperative complication due to this procedure. In 7 feet the implants were in situ due to open growth plate.
***Conclusion*** A common problem but not often mentioned problem of equines foot following club foot treatment is difficult to treat. Although temporary epiphyseodesis of the distal Tibia is a simple reproducible procedure, the results in our series are rather disappointing. There is not demonstrable correlation between the degree of correction (in degrees) measured on X-rays in the distal tibia to the gain in the dorsiflexion of the ankle joint measured clinically. The exact reason for the failure in improvement in the dorsal extension is not clear. Several causes must be here considered. There are several uncertainties with this treatment such as time of implantation, explantation, minimum tolerable ADTA angle, predictability of improvement in dorsiflexion and long term impact of such correction. Our study can only through an insight on this procedure as we are aware, that in some of our patients the correction is still in progress.

***Disturbed growth of spine/deformities of the feet V5.6***

### *Hallux Varus Congenitus and Hallux Varus Duplicatus*

E. Lamprecht*

Kantonsspital Winterthur, Kinderorthopädie, Winterthur, Schweiz

***Question*** Among the supernumerary toes—(polydactyly), hallux varus congenitus and hallux varus duplicatus take a special position.
***Methodology*** The diagnosis is made clinically, but treatment strategies are made in accordance with the radiological appearance and classification, which is made with radiographs, eventually in addition with MRI.
In comparison to polydactyly of the lesser toes, in hallux varus and hallux varus duplicatus correction means not only pure surgical excision of the duplicated part. An obvious deltaphalanx needs the excision of the bracket epiphysis; the varus position of the remaining first toe might need reposition of the phalanx in the metatarsophalangeal joint, possibly in combination with an osteotomy and Z-plasty of the skin.
The treatment is complex and should be reserved to the experienced surgeon.
***Result*** According to the classifications, the surgical techniques, depending on the different clinical and radiological appearances, are presented on our 8 patients.
***Conclusion*** Following the correct surgical strategy, the outcome is favorable, but secondary corrections might be possible during growth of the foot.

***Disturbed growth of spine/deformities of the feet V5.7***

### *Surgical Treatment of Rigid Cavovarus Deformity in Children and Adolescents*

S. Müller*, T. Dreher, K. Fehre

Department für Orthopädie und Unfallchirurgie Universitätsklinik Heidelberg, Sektion Kinderorthopädie/Fußchirurgie, Heidelberg, Deutschland

***Question*** The treatment of rigid cavovarus deformity in children can be challenging. The most common cause is Charcot–Marie–Tooth disease [1].Treatment options should be individualized and any bony procedure must be accompanied by soft tissue balancing. These patients must be monitored frequently during growth because further treatment may be required.
***Methodology*** 30 patients (10–17 years) with rigid cavovarus deformity (54 feet)received a reconstruction consisting Chopart arthrodesis, tibialis post. split transfer, osteotomy of the first metatarsal, plantar fascia release, Jones-procedure, long toe flexors tenotomy and if necessary AT lengthening. The radiographic evaluation consisted of standing a.p. and lateral views of the foot pre- and postop. The talo-first metatarsal (Meary), calcaneal-MT1 [1] and calcaneal inclination angles were measured on the lateral X-ray of the foot. The foot height/length ratio was calculated as the ratio of the height of the navicular to the length of the foot [2, 3]. The patients were monitored postoperatively twice a year.
***Result*** The correction of the cavovarus deformity was achieved in all patients. The average calcaneal inclination, talo-first metatarsal angle, navicular height:foot length ratio and calcaneal-first metatarsal improved. During the follow-up period (2.5–4 years) no significant recurrence was observed.
***Conclusion*** Rigid cavovarus deformity in children caused by CMT disease is rare. A careful preoperative evaluation is mandatory, thereby treatment should be individualized for each patient. Surgery using a variety of muscle balancing procedures and osteotomies are the treatment of choice for the progressive cavovarus deformity in younger patients. Goal of surgery is a well aligned fore- and hindfoot and balanced remained muscle power. For a patient with rigid deformities arthrodesis of talonavicular, calcaneocuboid and subtalar joint seems to be the only option.

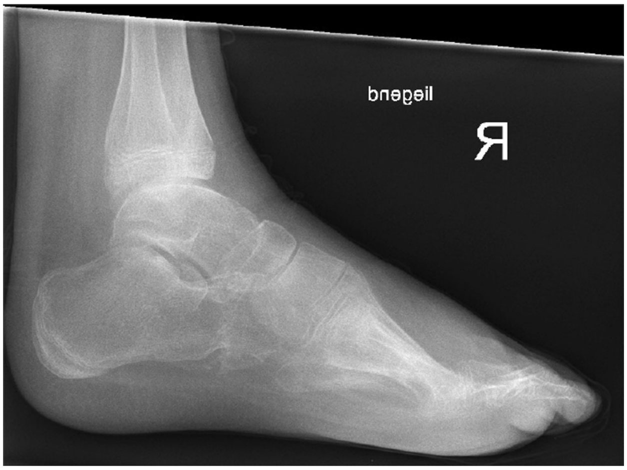

Post operative X-ray

***References***
[1] Aktas S, Sussman M (2009) The radiological analysis of pes cavus deformity in Charcot Marie Tooth disease. J Pediatr Orthop B 137–40
[2] Steel MW 3rd, Johnson KA, DeWitz MA, Ilstrup DM (1980) Radiographic measurements of the normal adult foot. Foot Ankle 151–8
[3] Saltzman CL, Nawoczenski DA, Talbot KD (1995) Measurement of the medial longitudinal arch. Arch Phys Med Rehabil 45–9

***Disturbed growth of spine/deformities of the feet V5.8***

### *Lengthening in Brachymetatarsia with an Internal Fixator*

I. Fichtel*, M. von Knobloch, P. Schmittenbecher

Städtisches Klinikum Karlsruhe, Kinderchirurgie, Karlsruhe, Deutschland

***Question*** The brachymetatarsia is a rare disease with an incidence of 2–5: 10,000. Boys are more frequent affected than girls (25:1). Nevertheless, more girls present with the desire of a surgical correction. However, not only the cosmetic aspect should indicate surgery, but also the possible development of metatarsalgia.
Two methods of correction are available: one step correction with bone grafting for lengthening up to 15 mm, and the gradual lengthening by callus distraction with an extern or intern fixator for a length discrepancy up to 28 mm.
***Methodology*** We use the internal fixator (type Genos MT Mini) for metatarsal lengthening. The interdigital handle for distraction is the only part outside skin level. Thus, a high wearing comfort is given to patients. After reaching the final length the handle is removed, leaving the fixator in situ until adequate bone consolidation.
***Result*** Since 2011 we carried out 7 metatarsal lengthenings for a total of 6 patients. The mean lengthening distance was 18.77 mm (8.93–27.75 mm) for an distraction period of 54 days on average. The average period until bony consolidation of the distraction callus was 307 days, with one patient still being in the consolidation phase. Thus, the average length of time of wearing the internal fixator was 360 days.
Complications such as infection or soft tissue problems by the fixator were not observed in our patients. In one case the extensor tendon was fused with the callous regenerate, so that a z-shaped tendon extension was required during metal removal. All patients were satisfied with the results despite the long duration of treatment.
***Conclusion*** Based on our experience, we consider the use of the internal fixator as the method of choice for the treatment of brachymetatarsia.

***Disturbed growth of spine/deformities of the feet V5.9***

### *Correction of Adduction and Supination Residual Deformity in Clubfoot*

C. U. Dussa*, F. Paulitsch, L. Doederlein

Orthopädische Kinderklinik, Kinderorthopädie, Aschau, Deutschland

***Question*** An adduction and supination deformity of fore foot is a well known deformity that can remain persistent following the correction of clubfoot either by Ponsetti regression technique or by peri-talar release. This deformity typically presents between 4 and 6 years. However deformities when uncorrected may worsen with growth. We present our experience to correct such deformities with Cole's Osteotomy.
***Methodology*** A study was done using the data from a prospective foot database. The inclusion criteria were: idiopathic clubfeet and clubfeet due to Arthrogryposis and minimum follow-up time was 2 years. Exclusion criteria were: Clubfeet due to other causes such as Meningomyelocele, Charcot–Marie–Tooth and loss of follow-up. The data collected were: demographics, diagnosis, type of surgery, use of bone graft, radiological union, complications, and clinical outcome.
***Result*** Twenty three feet in 21 patients were operated between 2010 and 2011 for residual forefoot deformity following clubfoot correction. One case (1 foot) was lost for follow-up. M:F = 13:8. The average age at the time of surgery was 11.6 years. Of the 21 patients 17 patients had idiopathic clubfeet; others had clubfeet due to arthrogryposis. In all cases a Cole's osteotomy was used to correct the pronation, abduction deformity. Correction of midfoot cavus deformity was done where necessary. Additional surgeries such as lateral shift osteotomy of calcaneus, transfer of Tibialis Anterior tendon, Augmentation of Achilles Tendon, supramalleolar Osteotomy were also performed where necessary. Complications included: intrinsic muscle palsy −1, painful dorsiflexion of ankle due to talo-tibial impingement −1. No case of wound infection, dehiscence or skin necrosis, delayed or non-union were noted. Plantigrade foot correction was achieved in all cases.
***Conclusion*** Residual deformity following clubfoot correction is usually in more than one plane. Such deformities cannot be optimally corrected by Mc Hale's procedure. Although this procedure can excellently correct a transverse plane (adduction) deformity, its ability to correct the forefoot supination is limited. Excessive plantar flexion of the medial column to correct severe supination leads to a medial column cavus deformity and may therefore reduce the dorsiflexion of the ankle joint.
These complex deformities may be corrected using Cole's osteotomy. It has a potential to correct midfoot deformity in all 3 planes. When carefully performed, complications are seldom.

### Infantile Tumour Surgery/Varia

***Infantile tumour surgery/Varia V6.1***

#### *The Delayed Diagnosis in Osteo- und Ewing Sarcoma: A Facebook-Based Survey*

M. Salzmann*[1], H. Rechl[2], R. von Eisenhart-Rothe[2], U. Lenze[2], F. Pohlig[2]

[1]Klinikum Schwabing, Kinderorthopädie, München, Deutschland, [2]Klinikum rechts der Isar, Klinik für Orthopädie, München, Deutschland

***Question*** The tumor size and existence of metastases at presentation are 2 of the most important prognostic factors in Ewing sarcoma and osteosarcoma. The earliest possible diagnosis is therefore essential to avoid an unfavorable prognosis. However, the average duration to diagnosis is still reported often with more than 3 months for both tumors. Due to the low incidence of these diseases, however, these data are usually collected over a long period of time and thus have limited actuality. Social networks like Facebook with their relevant groups especially for rare diseases such as sarcomas by the availability of a large number of affected persons represent a suitable medium for collecting such data. The aim of our study was to Internet-based survey of the duration of the first symptoms to histological diagnosis by means of an electronic questionnaire on Facebook.
***Methodology*** An electronic questionnaire with 10 items was created. A link to the online questionnaire was posted with the necessary information in special Facebook groups to osteo- and Ewing-sarcomas. After a month, the polls were closed and the data collected were analyzed statistically.
***Result*** A total of 27 patients were enrolled in our study online with osteo- and Ewing sarcoma. The average time to diagnosis was osteosarcoma at 2.92 and Ewing sarcoma at 3.27 months. The most common tumor location was the lower extremity in both entities. At the time of diagnosis, 83 % of patients with osteo and 73 % of patients with Ewing sarcoma were free of metastases. Surgical resection was performed in the osteosarcoma and Ewing sarcoma 100 % in 91 %. Chemotherapy underwent 92 % of osteosarcoma and Ewing sarcoma 100 % of the patients. Ewing sarcoma occurred in 55 % of cases additional radiotherapy.
***Conclusion*** Our study confirmed sarcomas long period of onset of symptoms to diagnosis with osteo- and Ewing sarcoma. Nevertheless, both entities had only a small proportion of patients at diagnosis on already detectable metastases. Furthermore, a social network such as Facebook could be successfully used as a modern medium in combination with an online questionnaire to collect epidemiological data of rare diseases in our study.

***Infantile tumour surgery/Varia V6.2***

#### *Primary Malignant Bone and Soft Tissue Sarcoma in Children and Adolescent: An Analysis of a Nationwide Sarcoma Center*

M. Salzmann*[1], F. Pohlig[2], H. Rechl[2], R. von Eisenhart-Rothe[2], N. C. Berger[1], F. Lenze[2]

[1]Klinikum Schwabing, Kinderorthopädie, München, Deutschland, [2]Klinikum rechts der Isar, Klinik für Orthopädie, München, Deutschland

***Question*** Primary malignant bone and soft tissue sarcoma constitute about 10 % of newly diagnosed cancers in children and adolescent. Pathologic diagnosis in bone and soft tissue tumors can be very difficult, whereas early diagnosis is crucial. The correct diagnosis and therapy requires a multidisciplinary team (radiologist, orthopaedic oncologist, pediatric oncologist and pathologist) to achieve the best possible results for the patients. Goal of the study was an analysis of the primary malignant bone and soft tissue sarcoma in children and adolescent, with special focus on diagnosis delay and outcome.
***Methodology*** This is a retrospective study design. Included were all children <18 years which were treated for a primary malignant bone or soft tissue sarcoma between 2000 and 2014. The clinical history, investigation and treatment were retrospectively analyzed with respect to localization, biology, age, duration of diagnostic setting, metastases, kind of operation and outcome.
***Result*** A total of patients 158 were included. 46 % had a diagnosis of an osteosarcoma and 33 % had a diagnosis of an ewing-sarcoma. Bone tumors accounted 86 % of the cases. The synovial sarcoma (33 %) was the most common soft tissue sarcoma. The most common localization was the femur (42 %), lower leg (21 %) and humerus (9 %). These preliminary results showed that the duration of diagnosis varied from a few weeks to several months. Limb preservation was the most common therapy for the tumors.
***Conclusion*** In this retrospective study bone tumors clearly outweigh the soft tissue tumors. The most common tumors were osteosarcoma, ewingsarcoma and synovial sarcoma. Early diagnosis and absent of metastasis is crucial for the outcome.

***Infantile tumour surgery/Varia V6.3***

#### *Influence of Pathological Fractures on the Prognosis of Primary Malignant Bone Tumors*

M. Schlegel*[1], M. Zeumer[1], S. Burdach[1], P.M. Prodinger[2], H. Rechl[2], I. von Luettichau[1]

[1]Division of Pediatric Hematology/Oncology, Wilhelm Sander Sarcoma Unit, Roman Herzog Comprehensive Cancer Center, Klinikum rechts der Isar, Technische Universität München, Department of Pediatrics, Munich, Deutschland, [2]Department for Orthopedic Surgery, Klinikum rechts der Isar, Technical University of Munich, Department for Orthopedic Surgery, Munich, Deutschland

***Question***: The purpose of this study was to investigate whether a pathological fracture has an effect on the prognosis of patients with osteosarcoma and Ewing tumor regarding 5-year survival, occurrence of metastases and local recurrence.
***Methodology***: We retrospectively analyzed 205 patients with the histological diagnosis of either osteosarcoma or Ewing tumor. Survival analysis was performed for all patients and differentiated for the following subgroups: osteosarcoma, Ewing tumor, adults and children.
***Result***: Out of all patients, 127 had an osteosarcoma and 78 had an Ewing tumor. The age ranged from 3 to 79 years (median age 17). 41 patients (20 %) sustained a pathological fracture. Patients with pathological fractures showed lower survival rates with 5-year survival rates of 64 % compared to 83 % ($p = 0.023$). The difference in development of metastases between patients with and without a pathological fracture was not statistically significant ($p = 0.286$). A local recurrence occurred in 7 % of the patients without and in 24 % with a pathological fracture ($p = 0.023$).
In subgroup analysis, there were no statistically significant differences between patients with and without a pathological fracture in survival and local recurrence rates for patients with Ewing tumor and in children. In contrast, the survival rate decreased from 83 to 59 %

(p = 0.024) and local recurrence increased from 70 to 87 % (p = 0.023) in the group with osteosarcoma. For adults, survival rates decreased from 78 to 51 % (p = 0.004) and local recurrence increased from 58 to 87 % (p = 0.001). None of the subgroups showed a statistically significant correlation between pathological fractures and metastases.

***Conclusion***: The present study suggests that the occurrence of a pathological fracture in primary malignant bone tumors has a negative effect on survival rates and implicates an increased risk of local recurrence in patients with osteosarcoma and in adults. In patients with Ewing tumor and in children, no prognostic effect of pathological fractures could be observed. The influence of pathological fractures on metastases did not reach statistical significance.

***Acknowledgments*** This work was supported by the Wilhelm Sander Stiftung.

***Infantile tumour surgery/Varia V6.4***

### *Morbus Trevor: Clinical Manifestation and Therapy of Dysplasia Epiphysealis Hemimelica*

C. Wack*, O. Eberhardt, F. Fernandez, T. Wirth

Olgahospital, Orthopädische Klinik, Stuttgart, Deutschland

***Question*** Dysplasia epiphysealis hemimelica or Morbus Trevor is a realy rare disease with an incidence of 1:1 million. The disease ist characterised with benign osteocartilaginouse tumors in epiphysis. The tumor occurs preferred at the lower limb, typically at one side of the epiphysis. This osteochondrom ist growing from the epiphysis and can destroy the joint area, so it is differently to the cartilaginous exostosis. The disease lead to limitations in movement, pain, leg length differences and deformities. The etiology is until now unknown. Up to date there is no evidence of a hereditary genesis. The relation of boys to girls amount to 3:1.

A statement respective frequency of development of malposition and there consequences is not possible so far.

We report about 12 cases in our department with regard to clinical symptoms, therapy and progress.

***Methodology*** Between 1999 und 2014 we examined 12 patients with a dysplasia epiphysealis hemimelica. The classification result from Azouz et al.

Examined were initial symptoms, classification, affected joint, side, age, therapeutic intervention, progress and complications.

***Result*** We see 8 boys and 4 girls. The main age was 8 years at diagnosis, 3 children were diagnosed widely before.

In our 12 patients were 5 patients who had initial multiple symptoms. Primary symptoms were in over 50 % of the patients swelling, 2 patients indicated pain, a deformity was occurrence in 3 patients. A limitation of movement was seen in 4 patients.

Mostly pain was indicated at the age of 8–13. In two cases the diagnoses was an incidental finding after a sprain.

The talus was the most frequently localization, followed by the distal tibia.

In one case we saw an incidence of both sides at the femurcondyle, which could treated conservative. In all the other cases a surgical intervention was necessary, partly with follow interventions.

***Conclusion*** The dysplasia epiphysealis hemimelica (M. Trevor) is rare and shows an overshoot growing of osteocartilaginouse area in epiphysis. This run the risk of premature occlusion of epiphysis, malposition, destruction of the joint area and resultant prearthrosis.

Routine controls of the growth of patients with a M. Trevor are obligatory require, particularly until the end of growth, but also beyond that, to prevent deformities and prearthrosis.

***Infantile tumour surgery/Varia V6.5***

### *Osteomyelitis of the Patella: Rare Disease and Therefore Important Not to be Missed*

E. Lamprecht*

Kantonsspital Winterthur, Kinderorthopädie, Winterthur, Schweiz

***Question*** Osteomyelitis of the patella is mostly seen in young children. Because of its rarity and variety of presentation, the diagnosis may be missed or delayed.

***Methodology*** Besides peripatellar pain, often with acute exacerbation with impossibility to walk, the clinical signs are usually swelling, painful prepatellar bursitis and septic arthritis oft the knee.

Once the diagnosis is clear, treatment follows the strategies of osteomyelitis with evacuation of pus, debridement of the necrotic parts of the affected patella and irrigating the knee joint. Systemic and specific antibiotics are continued according to clinical improvement and decreasing C-reactive proteine.

***Result*** We present 3 cases of patellar osteomyelitis, the tools to get to the correct diagnosis, the treatment and the outcome .

***Conclusion*** Our aim is, to point to a rare but easily missed infectious disease. If correctly treated, the outcome is favorable.

Short survey over the literature.

## Poster Presentation

***Poster session P.01***

### *3 Entities: 1 Goal: Temporary Hemi-Epiphyseodesis to Stop Progression of Severe Varus Deformity. Reports and Review of the Literature for Morbus Blount, Multiple Epiphyseal Dysplasia and Focal Fibrocartilaginous Dysplasia*

H. Tretow*[1], B. Vogt[1], G. Gosheger[2], B. Schuhknecht[2], F. Schiedel[1], R. Rödl[1]

[1]Kinderorthopädie, Deformitätenrekonstruktion und Fußchirurgie, Universitätsklinikum, Münster, Deutschland, [2]Klinik für Allgemeine Orthopädie und Tumororthopädie, Universitätsklinikum, Münster, Deutschland

***Question*** In natural course of leg axis development, a slight varus is physiological in early years of childhood. Besides that, paediatric orthopaedic surgeons know numerous causes for pathological varus deformity in the proximal tibia. Among those, we find rare diseases such as Blount's disease, multiple epiphyseal dysplasia and the focal fibrocartilaginous dysplasia. These require surgical treatment beyond growth guiding by hemiepiphyseodesis.

***Methodology*** We report three different cases of paediatric orthopaedic patients with full-blown varus deformity of the proximal tibia which were symptomatic as well as progressive. The cases are a matter of Blount's disease (BD), a fairbank form of multiple epiphyseal dysplasia (MED), and the focal fibrocartilaginous dysplasia (FFCD). Lateral hemiepiphyseodesis was performed in each case. The intention was not to achieve correction of the leg axis deformity by guided growth but to prevent further progression. More invasive surgical treatment for axial correction to later points of time were inevitable part of the concept.

***Result*** By lateral hemiepiphyseodesis, a progression of varus deformity could be stopped in all three cases with even a slight improvement of −7° (FFCD, right leg), −1°/−3° (MED, left/right), sowie −3°/−1° (BD, left/right). In literature, there exist only few cases of FFCD, several of MED and many of BD. Published treatment options vary from "wait-and-see" for FFCD, which tends to resolve spontaneously, to correction osteotomies in various techniques for MED and BD. To our knowledge, a concept of consecutive combination of epiphyseodesis and later correctional osteotomy has not been published until today.
***Conclusion*** Guiding growth by hemiepiphyseodesis is considered a possible and useful treatment even in severe cases of valgus or varus deformity in the absence of damage of the physis. In these demonstrated cases a structural alteration of the physis in deed exists and leads to progressive deformity and axial deviations. The effect of growth guiding therefore is limited. Nevertheless, hemiepiphyseal arrest can be applied reasonably to stop deformity progression. General recommendation for further surgical treatment, which is certainly necessary for deformity correction, cannot be given in these specific situations due to the rarity of the described diseases.

***Poster session P.02***

### *3-Level en Bloc Spondylectomy T4-6 of a Spinal NOS Sarcoma G2 in a 7 Year Old Boy*

S. Bockholt*, T. Lange, G. Gosheger, A. Schulze Bövingloh, T. L. Schulte

Klinik für Allgemeine Orthopädie und Tumororthopädie, Universitätsklinikum, Münster, Deutschland

***Question*** Spinal NOS (not otherwise specified) sarcoma is a rare tumor entity in children. Bone sarcomas account for less than 1 % of malignant neoplasms. Treatment of choice in G2 NOS sarcoma is wide tumor resection followed by radiotherapy leading to a 5-year survival rate of about 70 %. In literature there is a lack of data on spinal NOS, especially in children, as well as on en bloc spondylectomies in young children, especially multi-level procedures.
***Methodology*** A case report of a 7 year old boy with G2 NOS sarcoma of the upper thoracic spine is presented.
***Result*** A 7 year old boy initially suffered from back pain without neurological deficits for 6 weeks. Spinal MRI and CT scans revealed an osteodestructive tumor at T4-6 and large tumorous soft tissue masses surrounding the spinal cord. Staging excluded any metastasis. After confirming the diagnosis of a NOS spindle cell sarcoma G1-G2 by biopsy and presentation of only low grade metabolic activity in PET-CT the interdisciplinary tumor board decided to go for wide surgical resection followed by radiotherapy without any chemotherapy. A two-stage resection was carried out. On day 1, a posterior instrumentation T2-8 with a titanium pedicle screw-rod-system was performed with right-sided hemilaminectomy T4-6 and bilateral osteotomies of costae 4-6, ligation of right-sided nerve roots T4-6 and posterior mobilization of the tumor. On day 2, a high left-sided thoracotomy was done and the en bloc spondylectomy T4-6 including attached ribs was completed via anterior-posterior approach followed by reconstruction of the anterior column using a Harms cage filled with autologous bone. Neuromonitoring was uneventful.
Histological investigation confirmed the diagnosis of a NOS spindle cell sarcoma G2 with a wide oncologic resection except for one location close to the dura being marginal.
The patient was mobilized with a brace and dismissed 3 weeks after surgery with normal wound healing. The tumor board confirmed the treatment strategy and adjuvant radiotherapy with 59.4 Gy was done. The patient was closely followed by MRI and CT. After a follow-up of 2 years he shows an excellent oncologic and functional outcome without tumor recurrence, metastasis, neurological deficit, pain or implant failure.
***Conclusion*** Multi-level en bloc spondylectomy in the upper thoracic spine in children with malignant tumors is possible with excellent oncologic and functional results. Treatment of these patients requires a multidisciplinary approach in a highly specialized centre.

***Poster session P.03***

### *A Limping Adolescent: Der Intressante Fall*

F. Traub*[1, 2], M. Heng[1], A. Griffin[1], P. Ferguson[1], J. Wunder[1]

[1]University of Toronto, Mt. Sinai Hospital, Orthopaedic Oncology, Toronto, Kanada, [2]Universitätsklinik Tübingen, Orthopädie, Tübingen, Deutschland

***Question*** An 17-year-man presented with a 1 1/2-year history of right thigh pain. He ambulated with an antalgic gait favoring the right leg. He had no history of constitutional symptoms such as fevers, chills, or night pain. A trauma was not recalled. The intensity of pain as unchanged since its onset; it occurred mainly with walking or physical activity and was improved with the use of NSAIDs. Previous treatment with physical therapy resulted in no improvement. There was no family history of medical illnesses; in particular there was no history of cancer or other musculoskeletal diseases. On physical examination, he was a well-appearing man in no acute distress. Musculoskeletal examination revealed his right lower extremity to have normal neurologic and vascular findings. No palpable mass or localized area of tenderness was present, and ROM of the hip and knee were normal, but showed an limping gait favoring the right leg. Radiographs and MRI were obtained. The remainder of his examination and complete blood count and inflammatory markers were normal.
Radiographs of the right femur showed a 4.6-cm long surface lesion involving the medial cortex of the proximal femur. The lesion was located approximately 3.5 cm distal to the lesser trochanter. It was a well-circumscribed radiolucent lesion with a narrow zone of transition and no internal matrix. There was well-organized, thick, periosteal new bone formation proximal and distal to the lesion. On MR images the lesion was confined solely to the cortical bone; there was no extension into the medullary canal. The lesion had a signal isointense to muscle on the T1-weighted sequence and hyperintense on the T2-weighted sequence.

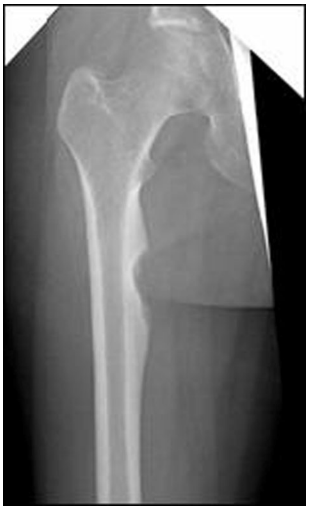

Radiograph right prox. thigh

The radiographic appearance of the lesion was consistent with a nonaggressive process.

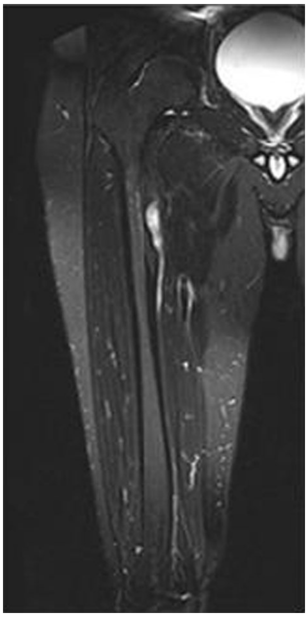

T2 right thigh

***Poster session P.04***

### *Age-Dependent 3D Foot Analysis During Heel-Raise of Paediatric Flatfeet*

B. K. Krautwurst*, S. van Drongelen, S. I. Wolf, T. Dreher, S. Müller

Universitätsklinik Heidelberg, Klinik für Orthopädie und Unfallchirurgie, Heidelberg, Deutschland

***Question*** Flatfoot is a common clinical finding in young children [1] and a frequent cause for orthopaedic consultation. In the typical examinations, heel-raise and Jack-Test [2], flexibility of the medial arch and position of the hindfoot are evaluated. Due to the subjective observation in these tests, a large variability concerning the interpretation and resulting treatment with regard to the child's age exists [3]. Hence, the aim of this study was to detect the age-dependent flexibility of paediatric flatfoot using an Methodology assessment, based on a 3D foot model.
***Methodology*** 33 children with flexible flatfeet and reference feet (mean age 7.2 years) underwent a 3D kinematic analysis of heel-raise, an analysis of foot pressure and a clinical examination. 9 markers were positioned on each foot and shank similar to a well described foot model [4]. The inclination of the medial border (movement of the medial border from medial to lateral) and the orientation of the heel (tilt of the heel in the frontal plane) were analysed. The right feet were divided into 3 age groups: 15 3–6 years, 12 7–9 years and 6 10–11 years. To compare the flexibility of the heel-raise between the groups, unpaired T tests were used at different positions of the angle between foot sole and ground.
***Result*** In all groups, the medial border changed from medial to lateral during the heel-raise (left figure). The young children demonstrated a higher dynamic, from increased medial to increased lateral inclination, compared to the middle-old children. The heel was corrected from a valgus position to varus (right figure). The young children showed an increased amplitude and dynamics compared to the middle-old children. In both movements, the old children demonstrated a similar curve progression as the middle-old ones.
***Conclusion*** The flexibility of the flatfeet was demonstrated in all groups, using the Methodology assessment. The medial border and heel lean were corrected during the heel-raise in each age group, however, the younger children showed a more dynamic movement and a higher amplitude. This study provides important Methodology information about the flexibility of paediatric flatfeet.

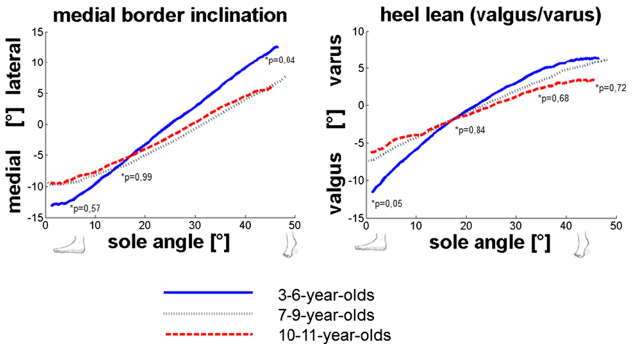

Medial border inclination and heel lean during heel-raise. P-values between young and middle-old group

***References***
[1] Pfeiffer M et al (2006) Prevalence of flat foot in preschool-aged children. Pediatrics 118:634–639
[2] Dare DM, Dodwell ER (2014) Pediatric flatfoot: cause, epidemiology, assessment, and treatment. Curr Opin Pediatr 26:93–100
[3] MacKenzie AJ et al (2012) The efficacy of nonsurgical interventions for pediatric flexible flat foot: a critical review. 32:830–834
[4] Simon J et al (2006) The Heidelberg foot measurement method: development, description and assessment. 23:411–424

***Poster session P.05***

### *Altered Rotational Leg Alignment in Flatfeet Predisposes for Knee Overloadings*

M. Hösl*, H. Böhm, C. Multerer, C. Dussa, L. Döderlein

Behandlungszentrum Aschau GmbH, Orthopädische Kinderklinik, Aschau im Chiemgau, Deutschland

***Question*** It had been previously documented that foot kinematics in flexible flatfeet [FF] are considerably abnormal [1]. Via the proximal kinetic chain, the deformity may yet also modify alignment of the tibia or femur and cause rotational joint stress or overloadings. We examined if pediatric and juvenile flatfootedness is related to the presence of transversal hip and knee rotation abnormalities during walking.
***Methodology*** In this cross-sectional study, 90 children and adolescents (age: 11.8 ± 2.7 years) with idiopathic FF from our outpatient clinic participated. 42 children with typically developed (TD) feet served as controls. Only the more involved side was incorporated. All participants performed a standard 3D gait-analysis (3DGA). Dynamic foot-progression, hip and knee rotations in stance were quantified and transversal knee moments calculated. Besides, 3D hind- and midfoot rotations were analysed with the Oxford Foot Modell (OFM).
***Result*** In comparison to TD, FF on average walked with the foot directed sign. more outward (+3°, P = 0.007). Kinematics in stance also revealed sign. more internal knee rotation (+5°, P = 0.017) and a more externally rotated hip joint (−3°; P = 0.026). The resulting rotation torque at the knee during early stance was 67 % higher than usual ($P < 0.001$). Correlations with the OFM data showed that the more supinated the forefoot when the heel strikes the ground (and the more subsequent pronating motion), the larger the knee rotation torque ($r = 0.34$, $P < 0.001$). The amount of rearfoot eversion was not correlated.

***Conclusion*** This shows that the adverse effect of FF is not limited to the foot but also alters transversal hip and knee rotations. Pathomechanically, subtalar pronation is thought to be coupled with internal tibia rotation which may position the patella unevenly in the femur [2]. This may induce longterm degenerations. Apparently, FF in adults were already associated with anterior knee pain [3], [4]. Also, more external hip rotation in pediatric FF has been reported [5]. Potentially, the hip attempts to balance the inward knee rotation. In practice, foot orthoses that accommodate forefoot supination in FF may help to reduce the rotational knee load. Generally, frontal plane forefoot alignment and flexibilty should be carefully examined in FF. Besides, without 3DGA, marking the patella may give first hinds for associated leg malrotations in FF.

***Poster session P.06***

### *Analysis of the Quality of Life in Children with Longitudinal Deficiency of the Lower Extremities, Arthrogryposis Multiplex Congenita or Skeletal Dysplasia*

A. Brockhaus*, D. Janßen, S. Nader

Schön Klinik, Klinik für Kinderorthopädie, Vogtareuth, Deutschland

***Question*** Patients with longitudinal deficiencies of the lower extremities, arthrogryposis multiplex congenital or skeletal dysplasia have different and often multiple physical handicaps. The aim of this study was to get valid datas about the self-perceived quality of life of the children themselves and the parents' perspective.

***Methodology*** 90 patients aged from 6 to 18 years with longitudinal deficiency of the lower extremities (proximal focal femoral deficiency, fibular deficiency, tibial deficiency or femor-fibular-ulnar-syndrome), arthrogryposis multiplex congenital or skeletal dysplasia were included in this study. Both the patients and their parents were asked to complete a questionnaire (ILK, University of Marburg, Germany). The problem-score (abnormal/normal) and the quality of life-score (above-average/average/below-average) were evaluated from the perspective of the patients themselves and from the perspective of the parents in comparison with a standardized population as mentioned in the ILK.

***Result*** In the group of patients with longitudinal deficiencies of the lower extremities the quality of life-score was average or above-average in 86–100 % (PFFD 92 %, 83 % of the parents; fibular deficiency 91 %, 96 % of the parents; FFU-syndrome 86 %, 100 % of the parents; tibial deficiency 100 %, 100 % of the parents). In patients with arthrogryposis multiplex congenita 89 % of the patients (88 % of the parents) rated the quality of life average or above-average. In patients with skeletal dysplasia this proportion was 95 % (100 % parents). The problem-score was normal in 67–100 % of the patients with longitudinal deficiency of the lower extremities (PFFD 83 %, 67 % of the parents; fibular deficiency 84 %, 91 % of the parents; FFU 67 %, 83 % of the parents; tibial deficiency 100 % of the patients and parents). In patients with arthrogryposis multiplex congenital this ratio was 85 % (80 % of the parents), in patients with skeletal dysplasia 94 % (88 % of the parents).

***Conclusion*** Patients with longitudinal deficiencies of the lower extremities, arthrogryposis multiplex congenital or with variant forms of skeletal dysplasia have a high proportion of average or even above-average quality of life from the perspective of the children/adolescents as well as from the parents' perspective in comparison with representative population data despite their physical restrictions. These results might contribute to a good clarification of young parents of a child affected by one of the above mentioned orthopaedic problems.

***Poster session P.07***

### *Anatomic Reconstruction of the Medial Patellofemoral Ligament in Children and Adolescents Using a Pedicled Quadriceps Tendon Graft*

M. Nelitz*[1], S. R. Williams[2]

[1]Kliniken Kempten-Oberallgäu, Akademische Lehrkrankenhäuser der Universität Ulm, MVZ Oberstdorf, Kempten, Deutschland, [2]Kliniken Kempten-Oberallgäu, MVZ Oberstdorf, Kempten, Deutschland

***Question*** Recurrent lateral patellar dislocation is a common knee injury in the skeletally immature adolescent. Because of the open physis, operative therapy in children is challenging. This study presents the outcomes of a minimally invasive technique for anatomic reconstruction of the medial patellofemoral ligament (MPFL) in children that respects the distal femoral physis and does not require hardware fixation at the patella.

***Methodology*** Twenty-three consecutive patients with patellofemoral instability and open growth plates underwent anatomic reconstruction of the MPFL using a pedicled quadriceps tendon graft. Preoperative radiographic examination included AP and lateral views to assess patella alta and limb alignment. Magnetic resonance imaging was performed to evaluate trochlear dysplasia and tibial tubercle-trochlear groove (TT-TG) distance. Evaluation included preoperative and postoperative physical examination, Kujala score, IKDC, Tegner activity score and activity rating scale.

***Result*** The average age at the time of operation was 12.8 years (range 9.3–14.1). The average follow-up after operation was 1.6 years after surgery (range 1.2–2.3). No recurrent dislocation occurred. The Kujala score significantly improved from 68.4 (range 47–81) preoperatively to 93.6 (range 76–100) postoperatively ($P < 0.01$). The median IKDC score improved significantly from 68 (range 44–84) preoperatively to 88 (range 70–98) at follow-up ($P < 0.01$). Three patients revealed a knee strength deficit 3 months after MPFL reconstruction, all three patients regained full strength at follow-up. The Tegner activity score and the ARS did not show significant changes. All patients were able to perform sports postoperatively.

***Conclusion*** The described technique for MPFL reconstruction in children and adolescents with a pedicled superficial quadriceps tendon graft is a safe and effective technique for the treatment of patellofemoral instability and allows patients to return to sports without redislocation of the patella. A major advantage of the quadriceps technique is that no hardware fixation or bony procedure involving the patella is necessary, making it ideal for use in children.

***Poster session P.08***

### *Anterior Knee Pain in a 15 Year Old Girl with a Hyperplastic Trochlear Groove and Femoral Neck Rotation Deformity*

S. Utzschneider*, C. Ziegler

Ludwig-Maximilians-Universität, Klinik und Poliklinik für Orthopädie, Physikalische Medizin und Rehabiliation, München, Deutschland

***Question*** A 15 year old girl presented to our ward with a history right anterior knee pain and a reduced range of motion, both of which had been persisting for several months. The symptoms were neither preceded by a traumatic event or dislocation of the patella nor the result of any known underlying condition. Sports were limited to physical education classes.
The physical examination showed slight knock knees, moderate effusion, distinct retropatellar crepitation, hypomobility of the patella and an extension capability reduced by 20° with free flexion.
MRI studies revealed 2nd to 3rd degree chondral lesions extending from the proximal apex of the patella over the lateral aspect all the way into the distal third of the patella. While the medial portion of the patella appeared to be dysplastic, the trochlear groove seemed to be deepened. Long leg radiographs showed a mechanical valgus of 5° for the right and 3° for the left leg. In the additional rotational computer tomography the femoral antetorsion angle was reduced to 3° on the right side. The left side was within normal range with 17°. Tibial torsion was 31° on the right and 33° on the left side, respectively.
A 3 month cycle of physiotherapy yielded an extension improvement of 5° but no pain relief could be achieved.
Since isolated cartilage repair would surely result in recurrence, we considered arthroscopic lateral release and a femoral rotation osteotomy mandatory to correct the reduced femoral neck antetorsion angle. Optional treatment could consist in coronal axis correction and trochleoplasty.
How to treat?

***Poster session P.09***

### *Arthroscopic Reduction and Primary Osteoplasty for Severe and Moderate Slipped Capital Femoral Epiphysis: Surgical Technique*

O. Hauschild*, A. El Tayeh, S. Herrmann, P. Helwig, N. Südkamp, K. Kuminack

Klinik für Orthopädie und Unfallchirurgie, Universitätsklinikum, Freiburg, Deutschland

***Question*** Slipped capital femoral epiphysis (SCFE) is the most common hip disorder in children and adolescents. In-situ fixation has traditionally been the treatment of choice even for severely displaced and unstable slips. As compared to mild slips long term results are, however, markedly impaired and high rates of avascular necrosis and early joint degeneration have consistently been reported. Moreover, more recent studies suggest that the residual CAM type deformity associated with SCFE may lead to early pathology at the acetabular rim cartilage and labrum.
The aim of this technical note is to present a novel approach that combines an arthroscopic reduction technique and screw fixation with primary osteoplasty in treating Southwick grades 2 and 3 slipped capital femoral epiphysis (SCFE).
***Methodology*** For this approach an open atraumatic reduction technique described by Gekeler in 2007 was adapted to an arthroscopic setting.
The procedure is performed under general anesthesia with the patient placed in supine position on a radiolucent table.
First a guide wire for a 7.0 mm cannulated screw is placed under fluoroscopic guidance via lateral stab incision and forwarded to the metaphyseal area. A 70 degree scope is then inserted into the peripheral compartment via the proximal anterolateral portal. Hemarthrosis is evacuated and an anterior working portal is established. Now, slight traction is applied to the leg by the assistant thereby widening the gap between the metaphysis and the acetabular labrum. A 3.2 mm threaded K wire is inserted into the joint via the anterior portal and placed through this gap onto the displaced epiphysis. Upon bony contact the K wire is drilled into the epiphysis. The epiphysis is now reduced against the metaphysis by manually pulling on the threaded K wire and simultaneous and coordinated traction and internal rotation of the leg. As soon as a satisfactory reduction has been achieved it is secured by advancing the prepositioned wire which in turn is exchanged for the cannulated screw. Now any residual CAM type deformity is treated by osteoplasty in a standard fashion.
***Result*** Thus far, ten children have been treated with this technique. No complications occurred and there was no case of AVN.
***Conclusion*** The present technique is a technically demanding but safe and feasible treatment option for SCFE. Longer follow-up and larger series are, however, needed to further evaluate this technique.

***Poster session P.10***

### *Biomechanical Evaluation of Two Tension-Band-Principle Based Tools for Guided Growth: FlexTack vs. Eight-Plate*

C. Druschel*[1], S. Hoppmann[2], R. Mayr[3], W. Schmölz[3], P. H. Pennekamp[2], R. Placzek[2]

[1]Charité-Universitätsmedizin Berlin, Centrum für Muskuloskeletale Chirurgie, Berlin, Deutschland, [2]Universitätsklinikum Bonn, Klinik und Poliklinik für Orthopädie und Unfallchirurgie, Bonn, Deutschland, [3]Universitätsklinik Innsbruck, Klinik für Unfallchirurgie, Innsbruck, Österreich

***Question*** Stevens et al. introduced in 2007 the eight-Plate to correct varus and valgus knee deformity and 1 year later also for correction of fixed knee Flexion deformity [1,2]. Nowadays the eight-Plate became gold standard for temporary hemiepiphysiodesis instead of the previously used Blount staples which after explantation may lead to epiphyseal plate compromise and overcorrection.
The purpose of this biomechanical study was to reveal differences in mechanical properties (of the tools) such as force absorbtion in relation to traction-induced displacement and ultimate failure including possible material failure during simulated epiphyseal growth.
***Methodology*** Forty fresh frozen porcine femurs were used by opening the epiphyeal growing zone with a thin chisel respecting the original anatomy of the epiphyseal line. In four groups with 10 femurs each a hemiepiphysiodesis was performed:

– SP-1: one hemiepiphysis in the sagittal plane with a 16 mm eight-Plate using 32 mm screws.

- SP-2: one hemiepiphysis in the sagittal plane with a 30 mm FlexTack.
- CP-1: two hemiepiphysis in the coronal plane with two 12 mm eight-plates using 24 mm screws.
- CP-2: two hemiepiphysis in the coronal plane with one 25 mm FlexTack.

The specimens were biomechanically tested using a servo-hydraulic material testing machine (858 Mini Bionix II, MTS, MN, USA) to determine strength at increasing displacement and failure load (N). For the statistical evaluation the software SPSS 21.0 (IBM Corporation, Armonk, NY, USA) was used. The significance level was determined at $p < 0.05$.

***Result*** For hemiepiphysiodesis in sagittal plane there were no significant differences found regarding Maximum load force (Fmax) and displacement at Fmax. for ventral hemiepiphysis a significant difference in the average maximum force as well as in the displacement at Fmax was found. The implanted CP-2 group showed a lower average maximum force and in particular a lower displacement at Fmax than the CP-1 group.

***Conclusion*** Ventral eight-Plate epiphysiodesis seemed to be superior in force absorption and fixation failure but a conclusion about a benefit in clinical use is not possible by this biomechanical data. Whereas due to the flat design of the FlexTack its implantation may cause less soft tissue irritation compared to the eight-plate.

***Poster session P.11***

### *Camptodactyly-Arthropathy-Coxa Vara-Pericarditis (CACP): Is There a Right Time for Surgical Treatment?*

J. Hewera*, R. Pasha

Helios Klinikum Krefeld, Kinderorthopädie, Krefeld, Deutschland

***Question*** We present a 14 years old girl with CACP. That is an extremely rare rheumatic disease characterized by non-inflammatory arthropathy with painful contractures of several joints and deformities of the bone in childhood and adolescence. This genetic disorder is caused by mutation in the Proteoglycan PRG4 gene on chromosome 1, which is a surface lubricant for joints and tendons. She and her two siblings suffer from CACP with massive contractures of joints and deformities in multiple regions. Nevertheless clinical problems like pain are given just in few joints. In X-rays there is also a pronounced hallux valgus, a pes excavatus and a lumbar hyperlordosis are seen. Moreover she presented a progressive and massive contracture of the hips and a coxa vara with cam impingement on both sides. The abduction of the hip joint was possible to 5° and very painful. After diagnostic arthroscopy of the right hip joint with partial synovectomy, capsulotomy, electrothermal denervation and abrasion of the cam she had a significantly improved movement of the hip joint without pain. Same procedure was done on the left side 3 months later with good result. As the possibilities of surgical treatment are limited with the risk of recurrences, it is not clear when operative intervention is recommended.

Does anybody have experience with operative results in patients with CACP? Is there a "right" time for surgical treatment? Which therapeutical steps are recommended?

***Poster session P.12***

### *Correction of Gait After Derotation Osteotomies in Cerebral Palsy: Are The Effects Predictable?*

H. Böhm*, M. Hösl, C. Multerer, C. Dussa, L. Döderlein

Orthopädische Kinderklinik, Kinderorthopädie, Aschau, Deutschland

***Question*** Derotation osteotomies of the femur and tibia are established procedures to improve transverse plane deformities during walking with inwardly pointing knees and in- and out toeing gait. However, effects of femoral derotation osteotomies on gait were reported to be small and those for the tibia are not known.

Therefore, the aim of the study was to show the relation between the amount of intraoperative rotation and the changes during gait for osteotomies at tibia and femur levels, and predict those for the femur from preoperative clinical and gait data.

***Methodology*** 51 patients with spastic cerebral palsy mean age 13.2 years were included, 46 limbs received rotation only at the femur, 6 only at the tibia and 11 limbs at both levels. Instrumented gait analysis and clinical testing was performed pre- and 20.6 ($SD = 1.9$) months postoperatively. The amount of derotation of femur and tibia measured intraoperatively, were correlated to the changes on hip rotation and knee rotation during gait, respectively. In addition a predictor analysis was performed from preoperative clinical and gait measurements

***Result*** The amount of intraoperative derotation of the femur did not correlate with the change in hip rotation during walking, whereas the rotation of the tibia showed an excellent relationship ($R = 0.84$, $p < 0.001$) with the change in knee rotation. Preoperative hip rotation during walking, passive hip abduction and foot progression angle together explained only 38 % of the variability of the postoperative change in hip rotation during gait. Strength and passive extension of the hip joint, and hip extension or abduction during walking did not show any predictive significance.

Prediction from preoperative measures may be only moderate because growth was shown to affect recurrence of internal hip rotation. This was confirmed, since adding femoral length change between pre and postoperative measures to the predictors increased the predictability from 38 to 49 %.

***Conclusion*** In conclusion changes of knee rotation during gait is directly predictable from the amount of tibial corrections, contrary the change in hip rotation was not related to the amount of femoral derotation, and prediction was only moderate. The predictor analysis revealed that small changes during walking can be expected when hip rotation is already close to the natural hip rotation. In addition, growth during the period until evaluation likely causes a loss of correction in hip rotation.

***Poster session P.13***

### *Correction of Procurvation Deformity near the Knee Joint in Children by Means of Guiding Growth*

L. Schwering*[1], V. T. Förster[2]

[1]Mathias-Spital, Technische Orthopädie, Rheine, Deutschland, [2]Universitätsklinikum Freiburg, Department Orthopädie Traumatologie, Freiburg, Deutschland

***Question*** The purpose of this evaluation is to demonstrate the results of correction of sagittal deformities near the knee joint by means of guiding growth in children. Guided growth with the 8-plate has proved as a valuable tool in the correction of deformities in the frontal plane near the knee joint like valgus or varus deformity. First P. Stevens reported about the initial experiences in the correction of procurvatum. This investigation demonstrates the first finalized results in the correction of paediatric procurvation of the knee joint by guiding growth.

***Methodology*** 20 extremities of 13 childrens were operated on for procurvation deformities near the knee joint because of spina bifida (7), achondroplasia (4), idiopathic deformity (3), cerebral palsy (2) infectious disease (1), muscular dystrophy (2) and transepiphyseal bar (1). The mean age at operation was 12 years and 4 month. The group consisted from 7 boys and 6 girls.

Therefore 22 8-plates were applied in the distal ventral femur while 14 8-plates were inserted in the proximal ventral tibia. In 11 extremities femur and tibia were treated simultaneously. The operative technique for the distal ventral femur was described by P. Stevens, while for the proximal ventral approach to the tibia one incision over the tuberosity was used to reach the epiphyseal plate medial and lateral from the tuberosity. Patients' files and radiographs were evaluated.

***Result*** Neither during the application nor during the hardware removal any complications occurred. In six extremities hardware removal was not necessary because of patienťs maturity. The 8-plate remained in situ for a mean duration of 12 months. The mean operation time/8-plate was 32 min. In 14 of the 20 extremities a full correction could be achieved while in 5 extremities the residual growth was too little to create a relevant correction in spite of having checked the epiphyseal plate by an MRI preoperative. In one case of posttraumatic deformity the situation remained unchanged.

***Conclusion*** Guiding growth seems to be a valuable tool in procurvatum deformities near the knee joint in children. Early intervention is necessary to avoid failure because of unexpected maturity. Because of the 3D structure of the growth plates near the knee K-wires instead of the screws have to be checked accurately by the intensifier. Potentially this indication will find a wider field in neuroorthopaedic diseases in the future.

***Poster session P.14***

### *Correction of Recurrent Equinus Deformity in Surgical Treated Clubfeet by Anterior Distal Tibia Epiphyseodesis*

N. Floeter*, K. Babin, S. Breyer, M. Rupprecht, K. Schelling, R. Stuecker

Altonaer Kinderkrankenhaus, Kinderorthopädie, Hamburg, Deutschland

***Question*** Since the wide spread acceptance of the Ponseti method, the relapse of clubfeet has become rare, still it is recorded to be 2–5 %. In contrast the recurrence of surgical treated clubfeet has been documented to be much higher, between 25 and 45 %. One problem is recurrent equinus deformity. Several treatment methods are described, mostly ending up in quite invasive surgical treatments such as osteotomies or soft tissue releases. As the procurvation of the tibia is one reason for the limited dorsiflexion in some patients, we want to evaluate the results of a minimally invasive procedure: the ventral epiphyseodesis of the distal tibia for treating recurrent equinus deformity in patients with elevated anterior distal tibia angle (ADTA).

***Methodology*** 20 children (age 8–14, mean 11 years), and 23 feet with pathologic ADTA were treated with ventral epiphyseodesis of the distal tibia with 8 plates from 2009 through 2014 in our institution. We evaluated them radiologically, measuring the ADTA and clinically, measuring the dorsiflexion of the ankle, preoperative and one to four times until removal of the 8 plate.

***Result*** At the time of abstract admission 11 patients (12 feet) have had removal of the plate ore closed growth plate. Their mean ADTA shifted from 86° (SD 2.1) to 74° (SD 6.5).The preoperative mean dorsiflexion of the ankle was 0.4° (SD 3.8) and improved to 6.3° (SD 4.6). Mean correction time was 19.4 (SD 6.4) months. The shift of the ADTA and of the dorsiflexion was highly significant ($p < 0.05$), but there was no correlation between the shift in clinical dorsiflexion and ADTA.

***Conclusion*** The ventral epiphyseodesis of the distal tibia is a valuable instrument for correcting recurrent equinus deformity in clubfeet with elevated ADTA. Nevertheless in some patients the dorsiflexion does not improve by shifting the ADTA. In our opinion there are other additional clubfoot related deformities like flat top talus or shortening of the achilles tendon which can be responsible for nonresponding in those patients.

***Poster session P.15***

### *Deformity Correction and Lengthening of the Lower Limb, 45 Surgeries in 24 Patients with Various Skeletal-Dysplasia*

J. K. Correll*, S. Nader

Schön Klinik, Klinik für Kinderorthopädie, Vogtareuth, Deutschland

***Question*** The skeletal dysplasias are a large, heterogeneous group of genetic disorders characterised by abnormal development and remodelling of the bones and cartilage. They typically present with short stature, disproportion, and various deviations of the lower limb. Significant advances in the non-surgical treatment of skeletal dysplasia have been achieved during the past years. Although extensive limb lengthening and deformity correction surgery is still an important part of the orthopedic treatment.

***Methodology*** We report the results on 45 deformity correction and limb lengthening surgeries in 24 patients with skeletal dysplasia (29 tibial/16 femoral lengthening). In this study only patients with chondrodysplasia punctata, pseudoachondroplasia, achondroplasia, metaphyseal dysplasia, larsen-like syndrome, SEDC and Phosphatdiabetes were included. The amount of deformity correction, external fixation time, length gained, mobility of adjacent joints, and incidence of complications were examined.

In 40 cases correction was done with the Taylor Spatial Frame. In 5 patients the Monolateral Rail System was appropriate. Bifocal osteotomy was necessary in 6 cases. The average age was 14.2 years, the mean follow up 2.4 years.

***Result*** The average correction time was 160 days (90–277 days). The mean duration of external fixation was 240 days (124–519 days). The mean deformity corrected was 28.9°, the average lengthening was 450 mm.

Major complications requiring surgical treatment were seen in 3 cases. In 1 patient with hypochondroplasia and in 1 patient with achondroplasia we had to stop tibial lengthening because of poor bone formation. In 1 patient with SEDC valgus deviation recurred after 1 year, temporary hemiepiphyseodesis was performed. There were 2 cases of subluxation of the knee. Because of technical limitations, osteotomy couldn't always cross the CORA, and translation was accepted. In all examined patients translation regressed with weightbearing after 1 year.

***Conclusion*** Also in these patients gradual deformity correction and limb lengthening using the Taylor Spatial Frame is a reliable method with low complication rate. To determine the right time and amount of deformity correction finding an accura tediagnosis is important to predict final height, expected complications and treatment as well as extraskeletal changes. In most patients with skeletal dysplasia the deformity is very complex and caused by intrinsic and extrinsic factors which should be considered before treatment.

***Poster session P.16***

### *Delayed Severe Case of Perthes Disease*

G. Salameh*

Waldhofzentrum, Kinderorthopädie, Kronberg, Deutschland

***Question*** Severe case of neglected Perthes disease, boy 9 years old with pain and severe limping.
Radiological dislocation and deformed flattened femoral head, pelvic obliquity and static scoliosis
***Methodology*** how to treat??
***Result*** Application of external fixation, distraction of hip joint, second stage salter osteotomy and reduction of femoral head.
Third stage pelvic support osteotomy.
Alternative treatment options??
***Conclusion*** New method in treatment of neglected perthes disease with use of external fixation.

***Poster session P.17***

### *Different Gait Patterns in Patients with Femoral Rotational Malalignment: Influence of Gait Analysis on Treatment Decisions*

M. Pallamar*[1], A. Kranzl[2], D. North[2], N. Wasserburger[2], R. Ganger[1]

[1]Orthopädisches Spital Speising, Kinderorthopädie und Fußchirurgie, Wien, Österreich, [2]Orthopädisches Spital Speising, Labor für Gang- und Bewegungsanalyse, Wien, Österreich

***Question*** Increased femoral antetorsion and femoral retroversion are associated with gait rotation malalignment and often require derotation osteotomies to restore anatomy. In symptomatic adolescents with abnormal rotational alignment additional assessment is done by CT or MRI and 3D-gait analysis. The purpose of this study was to compare static and dynamic alignment in these patients.
***Methodology*** 32 patients (21 female, 11 male, mean age 11.3) were included retrospectively. In these patients rotational malalignment based on CT (antetorsion angle <10° or >25°) was assessed in overall 55 limbs (in 2 patients only the left side, in 7 Patients only the right side). Additional 3D-gait analysis was done within 2 months prior or after CT. Antetorsion and tibia rotation data based on CT scans were compared to mean rotation values of the pelvis, the femur and tibia over single stance phase.
***Result*** 19/55 limbs showed increased femoral antetorsion with a normal tibia torsion (10°–30°) according to CT scans. In all these legs gait analysis revealed a normal femur rotation based on a dynamic muscular compensation. In two legs an additional pelvis external rotation was present.
Based on CT scans 24/55 legs had a rotational malalignment syndrome (femoral antetorsion >25° and external tibia torsion >30°). 13/24 legs showed a gait pattern with femoral internal rotation and tibial external resulting in kneeing-in gait. Active muscular compensation in the hole leg and pelvis was assessed in 9/24 legs. In two legs an external rotated pelvis was combined with a complete dynamic rotation compensation.
8/55 legs showed increased femoral antetorsion and reduced tibia external rotation (<10°) on the CT scans, gait analysis revealed in all 8 cases a muscular thigh compensation combined with a reduced or normal lower leg external rotation.
3/55 legs showed a reduced femoral antetorsion (relative femoral retroversion) on CT and had external thigh rotation at gait analysis.
1/55 with femoral retroversion on CT showed a complete muscular compensation with a normal rotation alignment profile in stance phase while walking.
***Conclusion*** Our results revealed a high number of muscular compensated gait pattern in patients with femoral rotational malalignment. This fact influences our patient-selection for derotation osteotomy.

***Poster session P.18***

### *Differential Therapy in Infants with Positional Skull Deformity*

H.-J. Kirlum*

Kinderchirurgie und Kinderorthopädie in der Au, München, Deutschland

***Question*** What are the risk factors for the development of a positional skull deformity? How effective is the therapy with or without a moulding helmet?
Since the “back to sleep” campaign more and more children show remarkable deformities of the skull. Depending on the threshold value up to 50 % of the infants were affected. The development of the head shape depends on the growth capability and growth pressure of the brain. Resent studies showed the possible impairment of the neurodevelopment in deformational plagiocephaly. This emphasises the importance of an effective therapeutic concept.

The therapeutic concept includes the information of the parents about the importance and the technique of positioning the baby, physiotherapy, osteopathy, treatment of cervical spine blockage and in some cases a moulding helmet. Depending on the age at the first presentation and the severity of the deformity the different recommendations were given.
***Methodology*** Including the anamnestic parameters, about pregnancy, birth, positioning, physiotherapy or other therapies, reflexes, mobility and the visual aspect of the skull deformity were documented. The head shape was measured with a 3D-imaging system. Anamnestic parameters, therapies, wearing time of the helmet, cephalic-index (CI) and the cranial vault asymmetry index (CVAI) were selected for further computations. The data of the patients were evaluated by the Department of Statistics of the Ludwig-Maximilians-University Munich.
***Result*** Risk factors for a skull deformity are caesarean section, vacuum extractor, multiples, preterm birth and cervical spine blockages. The CI and the CVAI in all children without a helmet showed a slight improvement, but the variability of the values was very high. The improvement of the CI and the CVAI shows a high correlation with the helmet therapy ($p < 0.0001$) and the age at the beginning of the helmet therapy ($p < 0.05$). The wearing time showed also a direct correlation with the improvement of the CI and the CVAI, but was not significant.
***Conclusion*** The nonsynostotic skull deformities are a challenging problem for the babies, their parents and the paediatricians. The structural development of the brain and the possible impairment of the neurodevelopment require a specific and individual therapeutic concept. The moulding helmet is an important part of this concept. To what extent the helmet therapy can support the neurodevelopment is goal of further studies.

**Poster session P.19**

### *Disabling Muscular Weakness and Massive Genua Valga Due to Rickets Secondary to Coeliac Disease*

M. Tedeus*

Kantonsspital Winterthur, Kinderorthopädie, Winterthur, Schweiz

***Question*** Severe rickets are disease only rarely seen in our everyday work. Presenting our case, we want to point out the aetiology and symptoms of this disease and sharpen our minds to them.
We are presenting the case of a 17-year-old Syrian girl with Trisomy 21. She suffered from diarrhoea, generalized joint pain, muscular weakness and severe genua valga. As a result, she was increasingly unable to walk and became dependent on a wheelchair.
Following extensive diagnostics, in the beginning suspecting a rheumatoid arthritis, we found severe rickets as the cause of these symptoms.
Thanks to gluten-restrictive diet and substitution of Vitamin D and Calcium, the patient soon became painfree, regained muscular strength and is now able to walk again.
Surgical correction of the genua valga will be performed when the nutritive status has been completely normalized.
Rickets are a rare cause for joint pain, muscular weakness and axial deformities.
Our case emphasizes the importance of the "thinking of" in everyday work.

**Poster session P.20**

### *Femoral Torsion in Children with PFFD (Proximal Focal Femur Deficiency)*

M. Klein*, H. Forkl, S. Nader

Schön Klinik, Klinik für Kinderorthopädie, Vogtareuth, Deutschland

***Question*** PFFD is a congenital rare disorder of the femur often associated with a length of leg discrepancy, malalignment of the axis and abnormity of the femoral torsion. A proximal focal femur deficiency often goes with a fibular hemimelia, instability of the knee joint and deformities of the feet. Due to this deformities affected children are dependent on orthopedic devices such as orthosis to compensate leg length inequality or to support the instability of the knee. There is hardly any literature concerning torsion measurement in children with PFFD. This study provides information about the ratios of the torsion of the lower extremities in children with PFFD.
***Methodology*** Between March 2011 and October 2014 eighteen children with PFFD underwent a torsion measurement with MRI or CT scan as well as a X-ray of both legs. The torsion of the acetabulum, femur and lower leg, the rotation of the knee and the instability index (Mc Kibbin Index) were evaluated as well as the length of femur and tibia. Measurement was done by the method of Schneider et al. 1997. 9 children were dependent on orthosis, 5 needed ortho-prosthesis and 4 of them had no orthopedic device.
***Result*** In 15 cases we carried out an MRI, in 3 cases a CT scan. The mean anteversion of the acetabulum of the affected leg was 12° (opposite side 17°), 7° anteversion of the femoral neck (opposite side 24°), 23° external torsion for the lower leg (opposite side 28°). The Mc Kibbin Index was 19 (opposite side 41). The femoral length measured 29.5 cm, the tibial length 27.3 cm (opposite side femoral 35.0 cm and tibial 28.0 cm). The total length of legs discrepancy was 6.3 cm. Three of the eighteen children received a derotation osteotomy after torsion measuring. 17 (94 %) of our children had an additional fibulahemimelia on the affected side.
***Conclusion*** Children with PFFD do have a reduced antetorsion of the femoral neck on the affected leg compared to the other side. According to our experience a derotation osteotomy simplifies the fitting of orthetic devices. A very high or low Mc Kibbin Index (30–40 normal) is associated with more pain and a higher rate of osteoarthritis. In our children the instability index is strikingly lower on the affected side compared to the other leg. This is another reason for us to run a torsion measurement for a correct planning of a derotation osteotomy. Our goal is to ease the supply with the orthosis and to improve the quality of life of children with PFFD by reducing the possibility of pain and osteoarthritis.

**Poster session P.21**

### *Gait Changes in Boys with Duchenne Muscular Dystrophy After Rideau's Multilevel Soft Tissue Release*

J. Funk*[1], C. Fritsche[1], A. Agres[2], S. Lebek[1]

[1]Centrum für Muskuloskeletale Chirurgie-Charité Universitätsmedizin Berlin, Kinder- und Neuroorthopädie, Berlin, Deutschland, [2]Julius-Wolff-Institut, Charité-Universitätsmedizin Berlin, Berlin, Deutschland

***Question*** Multilevel soft tissue release according to Rideau can prolong the ambulatory phase of boys with Duchenne muscular dystrophy (DMD). Its effect on gait has not been documented with instrumented gait analysis. The aim of this prospective cohort study is to analyze the results of Rideau's soft tissue release with regard to changes in spatiotemporal gait parameters and ground reaction forces.
***Methodology*** Boys who were eligible for Rideau's soft tissue release according to Forst's criteria were included in this study. Gait analysis was performed preoperatively, 3 and 12 months postoperatively. Standard spatiotemporal parameters including velocity, cadence, step length, walking base, minimal toe clearance, foot progression angle and double limb support were analyzed. Furthermore, maximal and total ground reaction forces were assessed. The results were normalized to account for changes related to growth.
***Result*** Between 05/12 and 09/13 5 boys with a mean age of 6.3 ($\pm$1.3) years at surgery were included in this study. None of the spatiotemporal gait parameters showed significant changes between the pre- and 3 or 12 months postoperative examinations. The same was found for the maximum anterior-posterior ground reaction forces, while the maximum vertical and medial forces decreased significantly after 3 months (1.34 vs. 1.20 and 0.18 vs. 0.10; $p = 0.043$) and did not show further changes between 3 and 12 months. Concerning total forces a significant post-operative increase (1.33 vs. 1.85; $p = 0.043$) was followed by a significant decrease (1.85 vs. 1.63; $p = 0.043$) during the 3–12 months period for the vertical component while the other directions showed no changes. Compared to an age and gender matched group of typically developing children the Duchenne boys walked with significantly lower toe clearance (1.49 vs. 0.68 cm; $p = 0.036$), a tendency towards slower gait (0.44 vs. 0.38; n.s.), broader walking base (0.16 vs. 0.23; n.s.), lower total vertical force (1.81 vs. 1.41; n.s.) and higher maximal medial force (0.11 vs. 0.18; n.s.).
***Conclusion*** The results of this study suggest that boys with Duchenne muscular dystrophy in this age group walk quite similar to their typically developing peers. When Rideau's procedure is done at this recommended point in time it has no negative effect on gait performance. Furthermore, it seems to have a stabilizing effect on gait for at least 1 year, which might explain its positive influence on the duration of the ambulatory phase.

***Poster session P.23***

### *How to Treat-Necrosis of Femoral Head and Neck Caused by Infantile Septic Coxitis*

D. Dovi-Akue*, P. Strohm, K. Kuminack, A. El Tayeh

Klinik für Orthopädie und Unfallchirurgie, Universitätsklinikum, Freiburg, Deutschland

***Question*** We present the case of a 7 year old girl suffering from femoral head necrosis due to septic coxitis of the right hip.
After an unremarkable pregnancy, childbirth and psychomotor development the girl came down with an acute septic infection by MRSA leading to a hematogenous coxitis at the age of 11 month.
The treatment performed in a neighboring hospital involved two lavages by arthrotomy, 6 weeks of i.v. antibiotics an 4 month of oral antibiotics. Follow up showed no recrudescence of the infection but the outcome of the coxitis was a necrosis of the femoral head with a stiff pseudarthrosis.
At the age of 2.5 years we performed a transplantation of a fibular graft into the femoral head. This couldńt prevent further progression of the femoral head necrosis and resorption of the fibular graft.
Actually the 7 year old girl walks with crunches and present a 3 cm shortening of the right leg, 15° extension lag and a possible abduction of 20°.
The radiograph shows a complete resorption of the femoral head and neck, the trochanter major is dislocated proximal to the acetabulum. We want to discuss possible treatment options like femoral head reconstruction, varisation and valgisation procedures in preparation for hip arthroplasty.

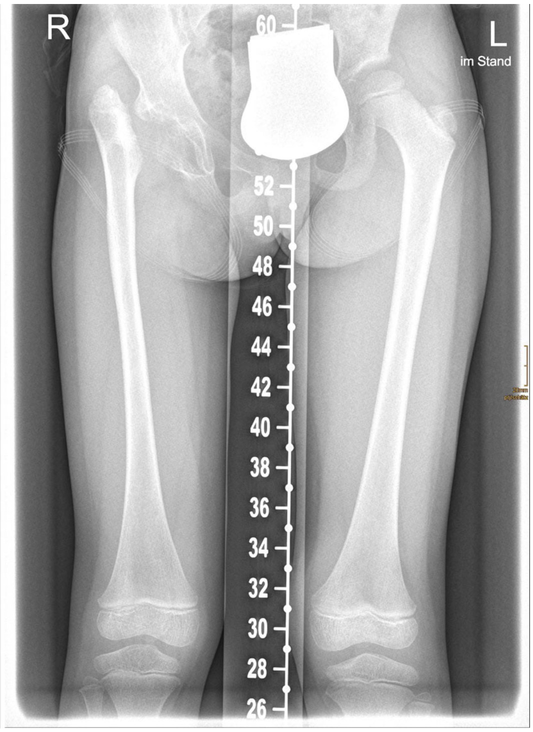

Current radiological status of the 7 year old girl with dexter necrosis of femoral head an neck

***Poster session P.25***

### *Hyperthyreosis: Uncommon Reason for Gait Disturbance in Orthopaedic Practice*

H. Lengnick*, H. Klima

Ostschweizer Kinderspital, Abteilung Kinderchirurgie, St. Gallen, Schweiz

***Question*** Reasons for gait disturbances might be multifaceted: next to frequent rotational problems of femur or lower leg in healthy children, neurological diseases, pathologies of the proximal femur like Morbus Perthes and Epiphyseolysis capitis femoris other reasons like endocrinologic diseases have to be considered.
***Methodology*** A 3-year old boy presented in the orthopedic ambulance with noticeable limping without pain. The parents reported restlessness of their boy, increased sweating during the day and mushy variation of defecation.
Excursion of the knee-joint during gait was strongly impaired, knee flexion during gait circle significantly reduced. Orthopaedic examination, almost impossible due to the boys‘ restlessness, showed normal ROM of the hip- and knee joints while muscle tension and tonus were markedly reduced. Ultrasound examination excluded the suspicion of a Coxitis fugax.
***Result*** Pediatric examination depicted emotional lability, enuresis diurnal and swelling of the eyelids. Laboratory examination showed pathologically increased T3 and T4 values while TSH was suppressed. Ultrasound examination showed pathologically enlarged, hypoechogenic glandula thyreoidea and secured diagnosis of hyperthyreosis.
Therapy with Carbimazol started to repress clinical symptoms during first 4 weeks of treatment.
***Conclusion*** Reasons for gait disturbances in children and young people might be multifactored. If classic peadiatric orthopaedic reasons for gait dysfunction can be excluded, also endocrinologic reasons have to be considered.
Hyperthyreosis influences oxidative phosphorylation leading to recuced ATP levels for muscle contraction causing muscle hypotonus. Exact anamnesis and clinical examination combined with interdisciplinary teamwork is helpful to secure this rare diagnosis in children.

***Poster session P.26***

### *If the Nutcracker Becomes a Brainteaser: Case Presentation of a Cuboid Fracture and Lisfranc Joint Injury in a 6 Year Old Boy*

H. Lengnick*, S. Christen, P. Gerhard, H. Klima, B. Schultz

Ostschweizer Kinderspital, Abteilung Kinderchirurgie, St. Gallen, Schweiz

***Question*** We demonstrate the case of a 6 year old boy presenting in the emergency department with foot pain after severe trauma by overrun of a 300 kg stone over his left foot.
***Methodology*** He was unable to walk due to pain on the left foot, we observed swelling of the lateral foot column and forefoot abduction. X-ray examination showed an impression fracture of Os cuboideum and fractures of Os metatarsale I–III. Dehiscence of the Metatarsale I and II basis >2 mm was suspect for a Lisfranc injury of the left forefoot and was confirmed by CT. Due to the instability and abduction deformity of the middle/forefoot, we decided for surgical intervention.

***Result*** A lateral approach and open reduction of Os cuboideum impression fracture was performed using the "Hintermann"-device to introduce a Tutobone wedge to hold reposition by K-wire osteosynthesis. Percutaneous fixation of Lisfranc joint was implemented using one intramedullare K-wire for Os metatarsale II and two K-wires for parallel Lisfranc joint fixation building the typical triangle. Postoperative after-treatment included plaster cast for 6 weeks without weight bearing, then K-wire displacement and another cast for 6 weeks while full weight bearing was allowed. Intraoperatively, the calcaneo-cuboid joint was completely reduced, but postoperative X-ray showed an oblique cuboid position in relation to calcaneus. Six week postoperative X-ray control showed the improvement of the oblique calcaneo-cuboid joint position while 12 weeks postoperatively the joint position was normal. An orthopaedic shoe inlay supported foot position of the medial and lateral foot column.
***Conclusion*** Cuboid-impression fractures are, in combination with Lisfranc joint injury, a very rare foot pathology in children and young people. The injury mechanism reminds of a nutcracker, when Os cuboideum is pinched between Calcaneus and the basis of Metatarsale V. Lisfranc joint injuries may lead to forefoot instability causing pain and longterm arthritis. Therefore surgical stabilization is necessary to prevent foot deformity, pain and abrasive wear. Literature describes only few cases with these kind of fractures in children or young people, but nutcracker and Lisfranc joint fracture have to be considered after severe foot trauma.

***Reference***
Ruffing T et al (2010) Die kindliche Nussknacker-Fraktur, 2010. Unfallchirurg, 113:495–500

***Poster session P.27***

### *Kniest Dysplasia*

D. Cerkez*, T. Wirth

Olgahospital, Orthopädische Klinik, Stuttgart, Deutschland

***Question*** Kniest dysplasia is a rare chondrodysplasia with mutation in the type II collagen gene, COL2A1. It is characterized by disproportionate dwarfism at birth, large head round face, flat midface, depressed nasal bridge, severe myopia, cataracts, vitreoretinal degeneration, retinal detachment, Cleft palate, hearing loss, recurrent otits media, short trunk, short neck, sternal protrusion, kyphoscoliosis, shortening and bowing of long bone, prominent stiff joints and clubfoot.
A 5 year old boy presented in October 2014 in our clinic. After birth the diagnosis of Kniest-dysplasia was made. He had been operated on his cleft palate. Because of his defective hearing he got hearing aids. He had also recurrent acute respiratory diseases after postnatal respiratory insufficiency. For his mobility he needs orthoses and other utilities. He could walk short steps free, for longer distance he needs a walking frame or he uses the therapeutic trainer bike.
Actually he is going into the regular kindergarten with integration help. He has twice a week physiotherapy with stretching and Galileo vibration. Now the family is consulting us and asking for better mobility.
Clinically the patient is mobile on his therapeutic trainer bike with ankle–foot orthoses. He can demonstrate a free walking but he manually supports his trunk. We see flexion contractures from both hips and knees. In supine position there is a flexion/extension 110-30-0° and an internal/external rotation 20-0-40°. The knee flexion/extension is 120-30-0. The ankle joint shows good mobility. The feet are in valgus position with syndactyly of digitus II and III. The spine is balanced but there is a small right convex scoliosis with hyperkyphosis and hyperlordosis. There is a limited spinal flexibility. On the upper extremity we see on both sides prominent elbow with extension/flexion 0-10-140°, the supination/pronation is 80-0-70°. On both sides we see ulna deviation of the wrist and the dorsal extension/palmar flexion 10-0-90°.
On the radiographic pictures there are bilateral coxa vara with broad metaphysis and missing ossification of both femoral heads and a dysplastic acetabulum. The feet are flat with ground-glass appearance of the bone, the talus is also flat. The spine demonstrates a small right convex scoliosis, hyperkyphosis and hyperlordosis with transformation of the vertebral bodies.
Treatment for better mobility in particular walking capacity?

***Poster session P.28***

### *Large Osteochondromas of the Femoral Neck: Results of Resection by an Intertrochanteric Osteotomy*

C. U. Dussa, C. Eisfeldt, S. Mehler, L. Doederlein*

Orthopädische Kinderklinik, Kinderorthopädie, Aschau, Deutschland

***Question*** Decentring of the hip or severe restriction of ROM in multiple hereditary osteochondromas (MHO) may cause considerable functional impairment through expanding dorsocaudal osteochondromas at the medial border of the femoral neck. Resection is necessary if pain and stiffness as well as radiographic signs of lateralisation of the femoral head occur. As the accessibility of these major bone masses through an anterior approach is difficult we have started to excise them through an intertrochanteric osteotomy approach. We would like to present our results and discuss the indication for resection.
***Methodology*** Between 2009 and 2014 we have treated 4 patients with MHO and functional limitations by an intertrochanteric osteotomy approach and resection of the bone masses. There were 2 girls and two boys 6,8,11 and 14 years old. All osteochondromas were on the left side. All osteotomies were internally fixed and the patients were treated by an early mobilization non weight bearing program for 6–8 weeks.
***Result*** All osteotomies healed uneventfully. The mobility of the hip joints could be re-established and the hips were concentric at latest follow up (6 months to 4 years postop.).There was no instance of AVN.
We indicated surgical resection when there were radiographic signs of decentration of the hip joint (widening of medial joint space) and when hip adduction, flexion as well as internal rotation were restricted to less than 0° of adduction, less than 90° of flexion and less than 0° of internal rotation, i.e. if an extension, abduction and external rotation contracture had developed. Pain was also an indicator for removal.
***Conclusion*** Removal of osteochondromas at the inferior border of the femoral neck seems indicated if progressive extrusion of the hip joint as well as restriction of hip ROM or pain occur. Removal through an intertrochanteric osteotomy approach is despite its invasiveness a safe method to remove all of the pathologically protruding bone by protecting the vascularity of the femoral head. Immediate mobilization is possible. Recurrence of the resected osteochondromas had not been detected until now.

***Poster session P.29***

### *Magnetic Controlled Growing Rods: First Experience in 12 Patients*

T. Lange*, S. Bockholt, G. Gosheger, A. Schulze Bövingloh, T. L. Schulte

Klinik für Allgemeine Orthopädie und Tumororthopädie, Universitätsklinikum, Münster, Deutschland

***Question*** Over the last years magnetic controlled growing rods have been introduced as an alternative to traditional growing rods [1] to facilitate distractions without any operative intervention. We report our experience in 12 patients.

***Methodology*** 12 patients have been operated within 18 months using magnetic controlled growing rods. The mean followup is 14.5 months (3–22 months). In these cases Cobb angle, angle of kyphosis (T4-12) and the distance between the spinous process of T1 and S1 were measured on X-rays pre-, postoperatively and at followup. Furthermore the number of distraction procedures, the total distraction distance, the number and type of revision surgeries and complications were analyzed.

***Result*** 12 patients (8 girls, 4 boys; type of scoliosis: idiopathic ($n = 6$), neuromuscular ($n = 4$), congenital ($n = 1$), syndromic ($n = 1$)) with a mean age of 10.4 y (6.8–15.9 years) have been operated before skeletal maturity using magnetic controlled growing rods. All patients received a dual rod construct. The fixation method varied between pedicle screws ($n = 10$) and rib and ilium hooks ($n = 2$) which were chosen due to dysplastic pedicles. On average 5 distractions (total 54, range 1–8) were carried out on a 3-monthly basis and 4.3 mm distraction could be reached at each time (Methodology: 5 mm).

Mean preoperative cobb angle was 72° (52°–109°), after surgery 41° (26°–72°, 43 % correction, $p \leq 0.001$) and at followup 41° (28°–93°, 43 % correction, $p \leq 0.001$). Mean preoperative kyphosis angle was 36° (17°–70°), after surgery 25° (2°–63°, 31 % correction, $p \leq 0.05$) and at followup 26° (5°–66°, 28 % correction, $p = 0.07$). On average the T1-S1 distance was 291 mm preoperatively (211-374 mm), after surgery 325 mm (253–414 mm, 11.7 % increment, $p \leq 0.001$) and at followup 335 mm (267–421 mm, 15 % increment, $p \leq 0.001$) resulting in a length growth T1-S1 of 8.3 mm/year.

In 2 patients implant associated complications occurred leading to revision surgeries (wound infection with subsequent removal of one rod, implant replacement after rod fracture).

***Conclusion*** It could be shown that the initial surgery using magnetic controlled growing rods leads to an acceptable Cobb angle correction which persisted during the following distractions. A kyphosing effect during distraction could not be detected.

This technique is safe and allows length growth over time of about 8.3 mm/year without any physically demanding consecutive surgeries. These results correspond well with the current literature [2].

***Poster session P.30***

### *Metamorphosis of Normal Human Lumbar Vertebrae to Quadruped-Like Shape by VEPTR Induced Growth Modulation*

C. Hasler*[1], D. Studer[1], P. Büchler[2]

[1]Universitätskinderspital beider Basel, Orthopädie, Basel, Schweiz, [2]Institute for Surgical Technology and Biomechanics Computational Bioengineering Group, Bern, Schweiz

***Question*** To assess the growth modulating impact of year long distraction-based VEPTR-treatment on vertebral morphology

***Methodology*** We digitally measured lumbar vertebral body heights (VBH) and upper endplate depths (VBD) at the time of the index procedure and at follow-up in 9 patients with rib-to-ileum constructs (group 1) spanning a normal lumbar spine. Nine patients with congenital thoracic scoliosis and rib-to-rib constructs but uninstrumented lumbar spines served as controls (group 2). All had undergone more than 8 half yearly VEPTR expansions. Wilcoxon signed-rank test was used for statistical analysis of initial VBH, VBD, height/depth ratio and at follow-up (significance level 0.05).

***Result*** The average age was 7.1 years (G1) and 5.2 year (G2, $p > 0.05$)) at initial surgery, the average overall follow-up time 5.5 years ($p > 0.05$). In both groups VBH increased significantly without significant intergroup difference. Group 1 did not show significant growth in depth, whereas VBD increased significantly in the control group. As a consequence H/D ratio increased significantly in group 1 whereas it remained unchanged in group 2. The growth rate in mm/year was 1.4 (group 1) and 1.1 (group 2, $p = 0.45$) for height and -0.3 and 1.1 ($p < 0.05$) for depth, respectively.

***Conclusion*** VEPTR growth modulating treatment alters the geometry of vertebral bodies by increasing the height/depth ratio. We hypothesize that in parallel to the distraction-induced gain of vertebral height, the implant related deprivation from axial loads (stress-shielding) impairs anteroposterior growth. The biomechanical consequence of such slender vertebrae exposed to unprotected loads in case of definitive VEPTR removal at the end of growth is uncertain.

***Poster session P.31***

### *Midfoot Joints Compensate the Decreased Dorsal Extension of Ankle Joint Following Clubfoot Correction*

C. U. Dussa*, F. Paulitsch, H. Böhm, M. Hösl, L. Doederlein

Orthopädische Kinderklinik, Kinderorthopädie, Aschau, Deutschland

***Question*** The commonest residual deformity following successful correction of clubfoot is reduced dorsal extension of the ankle joint. This may be due to several reasons. We undertook a study to see the effect of decreased dorsal extension in ankle on the foot.

***Methodology*** The data for the study was acquired from a prospective Gait laboratory database for clubfeet. All of them underwent foot analysis using Oxford foot model to assess the kinematics and kinetics. Inclusion criteria were: clubfoot of idiopathic aetiology, post correction through surgery, hind foot eversion. Exclusion criteria were: neurogenic and syndromal clubfeet, hind foot inversion. In all 58 patients were identified which fulfilled the above criteria. The observed parameters were dorsal extension of the ankle joint, dorsal extension of the midfoot, supination of the forefoot, various measurements of the Talus on X-rays. Statistical analysis was done to determine the significance of individual parameter.
***Result*** 48 Patients with 67 feet were included in the study. Of these, 19 patients had bilateral clubfoot. The compensatory mechanisms identified in the foot were increased dorsal mobility in the midfoot especially the increased dorsal extension in the midfoot joint and increased supination. Statistical analysis showed a strong correlation between decreased ankle joint dorsal extension and increased dorsiflexion of the midfoot joints. However no statistical significance was found between the decreased ankle joint dorsal extension and maximal supination of the foot in mid-stance and terminal stance phase. The measurements of bony landmarks identified the possible cause of decreased dorsal extension.
***Conclusion*** The effect of decreased ankle joint motion on the foot in clubfoot has not been mentioned. Dorsal extension of the ankle joint is necessary for 2nd Rocker motion during gait. Absence of the 2nd Rocker leads to premature heel raise. This is to some extent compensated by increase in midfoot joint motion predominantly dorsal extension. This finding has 2 clinical implications: (1) clinically measured passive dorsal extension of ankle joint is mostly the summation of dorsal extension in ankle and midfoot joints, (2) it is important to be aware of this compensation before one considers to fuse the midfoot joints to correct a midfoot deformity. This may lead to increased loading (impingement) of ankle joint and pain.

***Poster session P.32***

### *Mobility Without Wheelchair Despite Complex Dysmorphia-Syndrome*

S. Kappl*[1], M. Reiter[2], S. Nader[1]

[1]Schön Klinik, Klinik für Kinderorthopädie, Vogtareuth, Deutschland, [2]Spörer Gesundheitsgruppe, Vogtareuth, Deutschland

***Question*** Patients with complex defects of the lower extremities are obviously massively limited in their mobility. Insufficient operative treatment or rash amputations frequently result in difficulties of orthoprothetic care/supply. Therefore sufficient orthoprothetic treatment is facing a permanent wheelchair supply. In our case the positive effects of psycho-social development are impressivly shown but also the limits of technical ability by growing demands.
***Methodology*** A 14-year-old female patient with tibial and Patella aplasia and congenital dysplasia of the hips. In childhood Fixateur externe and verticalization by wearing ortheses. Attending elementary school for mentally and physically disabled where not challenged enough. A sufficient orthoprothetic treatment lead to integration in a normal school resulting in a normal maturation. The requirements of taking part in daily working life are provided. Growing demands in treatment due to free mobility, height, physical education and use of public transport. Main problems caused by worn out and insufficient fit of the ortheses due to loss of weight of 7 kg within 6 months. Showing pain in the rudimentary knees. Modification of ortheses: Femoral part with dorsal fixation and tube framing, knee flexible, tibial part with ventral fixation and extension, ring-like framing oft he foot, soft liner and carbon foot. Reducing weight by using carbonized GFK and no cosmetic lining.
Result The patient is walking without any aids apart from ortheses, even stairs in alternating pace, normal height. Problems in stabilizing the rudimentary knee joints. After calculating the costs the health insurance is in doubt of necessity of ortheses.
***Conclusion*** Continuously use results in short intervals of maintenance due to safety aspects. Several soft liners are necessary for changing. The main focus is put on stabilising the knee joints. The treatment is team work, the use of new technologies inevitable. Continuous new developmenst have to be proofed like the Sinterplot-processing. Tough the manufacture of parts is possible to do out-patient the actual testing has to be done under close therapeutical supervision i.e. in-patient. It has to be fought for the preservation of this option despite the growing costs. The staff might be stressed too much if the syndromes are exceptional. Discussions are helpful. In the end a doctor should review the result to ensure the quality and to optimize the process.

***Poster session P.33***

### *Multiple Osteochondromata (exostoses) in the Forearm with Carpal Deviation*

J. Willers, E. Dingeldey*, J. Matussek

Orthopädische Klinik Universität Regensburg, Kinderorthopädie, Regensburg, Deutschland

***Question*** Seperate lengthening processes of the deformed ulna in a twisted and deformed forearm yields a better hand function—cases presentation

***Poster session P.34***

### *Musculoskeletal Manifestations in Mucopolysaccharidosis Type I (Hurler Syndrome) Following Hematopoietic Stem Cell Transplantation*

S. Breyer*[1], M. Schmidt[2], U. Löbel[3], S. Yarar[4], R. Stücker[1], K. Ullrich[2], I. Müller[5], N. Muschol[2]

[1]Altonaer Kinderkrankenhaus, Kinderorthopädie, Hamburg, Deutschland, [2]UKE, Pädiatrie, Hamburg, Deutschland, [3]UKE, Department of Diagnostic and Interventional Neuroradiology, Hamburg, Deutschland, [4]UKE, Department of Trauma, Hand and Reconstructive Surgery, Hamburg, Deutschland, [5]UKE, Clinic of Pediatric Hematology and Oncology, Division for Stem Cell Transplantation, Hamburg, Deutschland

***Question*** Hematopoietic stem cell transplantation (HSCT) is the treatment of choice in Hurler patients (mucopolysaccharidosis type I-H, MPS I-H). Following HSCT life expectancy improves significantly, whereas musculoskeletal manifestations are assumed to be progressive. Better understanding of the underlying pathology of joint and bone disease becoming more relevant. The present study summarizes the progression of musculoskeletal manifestation in 19 Hurler patients following HSCT and proposes diagnostic algorithms and treatment recommendations.
***Methodology*** Range of motion (JROM) of shoulders, elbows, hips and knees in 19 MPS patients, who underwent allogenic HSCT between 1991 and 2012, were assessed during a mean follow-up period of 6.4 years (range 0.7–22.5 years). Radiographs of thorax, spine, pelvis and hands as well as magnetic resonance imaging (MRI) scans of the craniocervical junction (CCJ) and odontoid process were analyzed.
***Result*** Nineteen evaluable Hurler patients (10 females, 9 males) were included in the study. Ten patients achieved long-term engraftment with chimerism >94 %, seven patients presented with a mixed chimerism between 16.7 and 59.3 %, whereas two patients developed graft failure during follow-up. Ten patients developed acute Graft-versus-Host Disease (aGvHD). All patients had joint contractures. The majority of patients showed a steady state or even improvements in JROM of knees (33 or 56 % resp.), hips (41 or 35 % resp.) and elbows (56 or 33 % resp.) leading to a full range of motion in 39–61 % of joints. In patients with graft failure progressive restrictions in JROM were noted. Assessments of craniocervical stenosis (CCS) by MRI scan showed stable or improved diameters and correction of odontoid hypoplasia during follow-up in most cases and therefore no severe complications were recorded. However thoracolumbar kyphosis, genua valga as well as hip dysplasia were progressive despite HSCT.

***Conclusion*** HSCT does not only improve long-term survival and cognitive development, but has a beneficial effect on several musculoskeletal manifestations in Hurler patients. Nevertheless, frequent monitoring for skeletal complications and supportive therapies are essential to preserve and improve motor function.

***Poster session P.35***

### *Postradiation Acetabular Dysplasia in Rhabdomyosarcoma*

E. Dingeldey, J. Matussek*

Orthopädische Klinik Universität Regensburg, Kinderorthopädie, Regensburg, Deutschland

***Question*** Severe pain and leg length discrepancy in a 16 year old boy with acetabular dysplasia after childhood radiation. PAO and lengthening processes did not seem to have been sufficient. Muscular gluteal deficiency seems to produce major treatment difficulties.

***Poster session P.36***

### *Proximal Epiphyseal Slip Following Proximal Varus Derotation Osteotomy of Femur*

C. U. Dussa*, L. Doederlein

Orthopädische Kinderklinik, Kinderorthopädie, Aschau, Deutschland

***Question*** A 6-year old boy presented to our clinic with left sided hip dislocation. I was diagnosed to have Becker-Adams-Syndrome as a result of chromosomal anomaly.

Clinical examination showed an obese, non cooperative patient weighing 35 kg. A limb length discrepancy of 1.5 cm shortening on the left was seen. Passive hip abduction was reduced on the left side compared to right (R:L = 30°:10°). Otherwise all the other movements of the hip and knee joint are free and full. The ankle joints showed fixed equines deformity. In addition, the deep tendon reflexes were exacerbated. Clonus could be elicited. Babinski was positive on both sides. Walking with support was extremely cumbersome.

X-rays showed a left sided hip dislocation with acetabular dysplasia. The right hip was normal. A varisation, derotation shortening osteotomy (DVO) of the proximal femur was performed along with open reduction of the hip joint. A dega type of acetabuloplasty was performed. Postoperatively the patient was immobilised in plaster of Paris cast due to lack of cooperation. Following consolidation of the osteotomy in 6 weeks, he was referred to a rehabilitation unit. During mobilisation in a Locomat Trainer, he experienced pain in left hip which worsened, till the patient could no more be mobilised on Locomat Trainer. The rehabilitation was discontinued.

Examination on readmission at our hospital, showed elevated temperature 390 °C, increased leucocytes and CRP. X-rays showed osetolysis of the neck of femur with slip of the proximal femur epiphysis. An epiphyseal slip due to infection was suspected.

Arthrotomy showed blood stained fluid with no abscess. A valgisation osteotomy was done after reduction of the head and K-Wire pinning. Microbiology showed no bacterial growth. Follow-up after 5 years showed complete necrosis of proximal femur epiphysis with redislocation of the hip joint and severe hip dysplasia. On the right side as well, the hip showed a progressive hip dislocation.

***Discussion*** Literature shows only one publication with 2 cases describing similar chain events following surgery. The possible causes for this complication will be discussed. It is also to be discussed of the right hip now needs containment. The case has been presented for its rarity.

***Poster session P.38***

### *Reconstruction of a Traumatic Joint Defect in the Forefoot by Autologous Iliac Apophysis Transplantation in a Child*

L. Schwering*[1], V. T. Förster[2]

[1]Mathias-Spital, Technische Orthopädie, Rheine, Deutschland, [2]Universitätsklinikum Freiburg, Department Orthopädie Traumatologie, Freiburg, Deutschland

***Question*** So far only a few studies investigated techniques to repair joint and growth plate defects in the forefoot of children. Transplantation of autologous apophyseal cartilage has shown successful in reconstruction of joint defects in children in several case reports. These cases predominantly describe reconstruction of phalangeal joint defects in hand surgery.

How effective can apophyseal transplantation in forefoot reconstruction be?

***Methodology*** The case of a 6 year-old girl is subscribed who sustained a wound 2 × 2 cm in size over the medial part of the first metatarsophalangeal joint in an accident. Besides the extensive damage to the soft tissue and tendons the radiographs on admission revealed an osteo-cartilaginous defect of three-fourths of the proximal phalanx of the first metatarsophalangeal joint. After an initial debridement and using vacuum bandage for a few days we reconstructed the joint replacing the osteo-cartilaginous defect with an autologous cartilage graft of the iliac apophysis and tendon transfer. Then the residual soft tissue defect was covered with a rotation flap of the skin by our plastic surgeons. Due to an impaired healing of the skin flap revision surgery with transplantation of a skin mesh graft had to be performed 2 weeks later.

***Result*** Complete healing of the wound was achieved 6 weeks after the injury. Radiographs taken at 4, 6 and 12 months after surgical correction showed a good integration and remodeling of the grafted area. Full weight-bearing was allowed after 8 weeks. 6 months following surgery the child presented free from pain with an almost symmetrical range of movement in the first metatarsophalangeal joint comparing both feet.

***Conclusion*** We present the medium- term outcome transferring cartilage of the iliac apophysis to substitute a traumatic joint defect of the first metatarsophalangeal joint in a pediatric patient. To date the clinical and radiological result is satisfactorily 24 months after the injury. Of course the long-term result remains to be seen. In order to prove the efficacy of using non-vascularize autologous cartilage grafts in children to repair joint defects in the forefoot further trials with larger number of cases will be needed in the future

**Poster session P.39**

### Spinal Flexibility in AIS: A Non-Invasive, Pre-Operative and Patient-Specific Method

S. Berger[1], C. Hasler*[2], D. Studer[2], P. Büchler[1]

[1]Institute for Surgical Technology & Biomechanics Computational Bioengineering Group, Bern, Schweiz, [2]Universitätskinderspital beider Basel, Orthopädie, Basel, Schweiz

***Question*** Adolescent idiopathic scoliosis (AIS) is a three dimensional deformation of the spine. Surgery is discussed when thoracic curve is expected to reach at least 50° by maturity. To plan surgery and predict its outcome, spinal flexibility has to be evaluated preoperatively.

Several techniques are used clinically to evaluate spinal flexibility (side bending, fulcrum bending). All these methods use medical imagery to compare the shape of the spine before and after loading, but none report the amount of forces used to provoke the observed motion. Therefore, only reducibility (change of spine shape) can be determined and not the flexibility of the spine.

***Methodology*** For these reasons, an alternative pre-operative test has been developed. The Spinal Suspension Test is based on the application of an axial traction force on the patients' spines using a commercial head halter. The three-dimensional displacement of the vertebras is assessed with calibrated orthogonal radiographic images. The spinal shape is measured before and after application of a load corresponding to 30 % of the patient's body weight. A numerical model of the spine is proposed to quantify the patient-specific mechanical properties. The model consists of rotational springs to model the flexible components of the spine. Three linear spring were used for each segment to represent the spinal flexibility along the main anatomical directions: flexion, axial rotation and bending. The vertebrae were considered as rigid bodies. The boundary conditions correspond to the experimental test: the lower vertebra has been fixed while a vertical force was applied to the most cranial vertebra. The Nealder–Mead optimization algorithm was used to find the stiffness parameters which best match our experimental measurements.

***Result*** The Spinal Suspension Test has been applied on 5 AIS patients. Important variations of the stiffness parameters were observed between patients, while being comparable with previous intra-operative measurements.

***Conclusion*** Since the spinal shape and mechanical properties showed important variation across patients, this quantitative information provided by the proposed pre-operative test is critical for the development of planning solutions that consider patient-specific biomechanics. Such tools will become increasingly important in the future due to the ever-increasing complexity of the surgical instrumentation and procedures and to provide a better understanding of the complex biomechanical properties of the spine.

**Poster session P.41**

### The Indication of Genu Valgum Correction by Guided Growth

A. Schreiner*

Orthopädische Praxisklinik, Spaichingen, Deutschland

***Question*** Contrary to the genu varum we have not been able until now to identify the genu valgum as a precursor of osteoarthritis of the knee. How shall we counsel the families as to the question, how necessary the correction of knock knees is and to which extent the malalignment may be accepted?

***Methodology*** Literature research was carried out. Not only publications concerning the precise topic were considered, but also studies dealing with other subjects, but containing aspects of evidence, which would have a bearing on our questions. 110 publications were read and relevant evidence was extracted. The devolution of lateral knee OA cases in our own institution was checked against those literature results.

***Result*** The evaluation of the literature shows, that the genu valgum is not responsible for the occurrence of osteoarthritis of the knee. But it makes it quite clear that the progression of lateral knee OA is closely linked to knock knees [1, 2]. We were able to confirm this notion in our own adult patients [I1, I2]. This synopsis of the literature and our own documented experience also shows, that we must not simply accuse the forces of gravity to cause this progression of a lateral knee OA, but have to recognize complex processes, in which the iliotibial band plays a major role.

***Conclusion*** The genu valgum is not the cause of knee OA. But in case OA occurs its progression is facilitated by knock knees. Since this progression is a complex process, its occurrence follows stochastic rules. This means, in analogy to ionizing radiation, that the probability of an adverse event depends on the dose of the noxious agent, but not its severity. The tiniest exposure may have the same disastrous consequence as a massive dose, only with less probability. For this reason there can't be a threshold for the detrimental dose of the noxious agent. This means for the genu valgum, that a threshold for the noxious effect of the malalignment cannot exist and even a small valgus alignment entails the risk of progression of lateral knee OA. Therefore it is not reasonable to define a minimal extent of valgus deviation as indication of the correction. So we are in the position to advise the teenagers and their families in a conscientious way to have the correction done, even in case of a small extent of knock knees. But we have to admit that with this measure we shall not prevent diseases in adulthood. We are merely dealing with risks of disease aggravation.

**Poster session P.42**

### The Late Presentation of Knock Knees: A Case Report

A. Schreiner*

Orthopädische Praxisklinik, Spaichingen, Deutschland

There is a broad agreement that the development of leg alignment draws to a close approximately at 10 years of age. This means that after this age we need not worry about further aggravation of a malalignment. At the same time this is the age at which subtalar arthrorisis for a flexible flatfoot appears reasonable to be carried out. The upper age limit for this intervention is assumed to be 12–13 years.

We shall present the case of a boy, whose knock knees aggravated between the age 11 and 12.5 from a mild condition not requiring treatment to marked genua valga we plan to correct by guided growth.

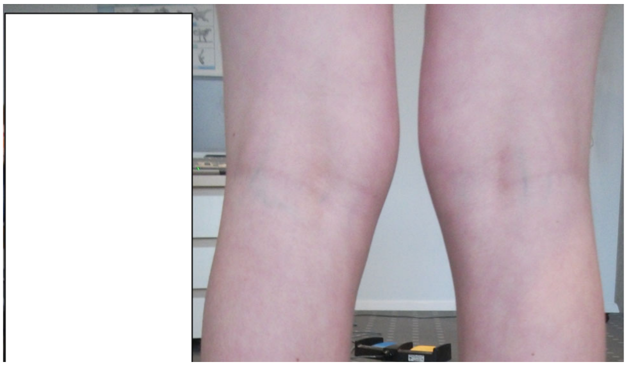

In the same period of time very mild flatfeet first aggravated to quite severe ones, as well, but resolved almost completely.

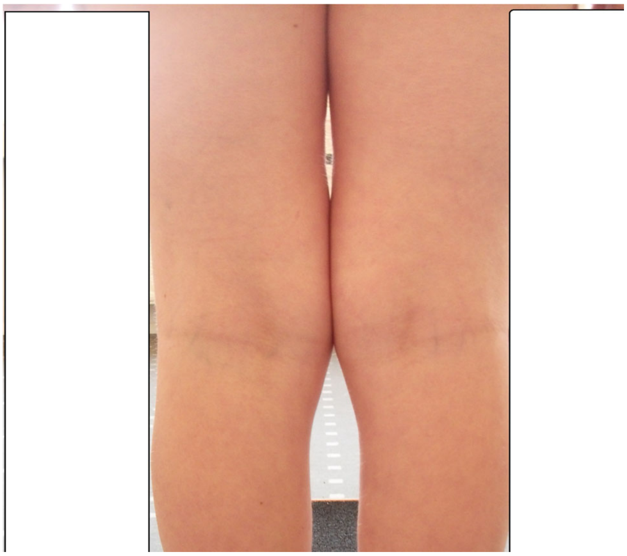

This case shows that the current concept of the development of leg alignment may not be necessarily correct. We are not save after the age of 10.
The case also shows that the operative management of a flexible flatfoot at the age of 12 might still be too early, because needless.
We recommend to follow the teenagers until near the end of growth with regard to leg alignment. As to the subtalar arthrorisis we consider 13 years rather an age to be reached before the decision of correction is being made than an upper limit.

***Poster session P.43***

### *The Results of Preoperative Halo-Gravity Traction for the Treatment of Severe Spinal Deformity in Children*

K. Mladenov*, R. Ivo, B. Erdinger, U. von Deimling

Asklepios Kinderklinik, Sankt Augustin, Deutschland

***Question*** Halo gravity traction has been used preoperatively for the correction of severe spinal deformity in children with good results. However the reported overall complication rate remains high and varies between 19 and 33 %.
***Purpose*** To assess the efficacy of our treatment protocol for preoperative halo gravity traction in children with severe spinal deformity with focus on complication rate.
***Methodology*** This is a single center, retrospective study, Seventeen pediatric patients with severe, rigid scoliosis and/or kyphosis of more than 100° underwent preoperative, gradual, transferable, continuous halo gravity traction (bed, wheelchair, walker) of up to 40 % body weight, applied for an average of 6 weeks according to our established treatment protocol. Data collected from patient records were analyzed with emphasis on complications. Deformity correction was evaluated using serial, standing X-rays (pre-treatment, at completion of traction and after surgery).
***Result*** Eleven patients underwent after completion of traction spinal Fusion and 6 were treated with a "Non-Fusion" technique. Mean Cobb Angle before traction measured 118° (100°–174°). Curve magnitude after completion of traction averaged 85° (46°–141°) and after surgical intervention—63° (32°–123°).
There were no neurologic complications. In only 2 Patients minimal pin irritation occured during traction which healed with local measures.
***Conclusion*** Halo gravity traction is a safe method for gradual correction of severe spinal deformity in children. Complication rate can be significantly reduced using standardized treatment protocol.

***Poster session P.44***

### *Treatment of Congenital Pseudarthrosis of the Tibia Using BMP-2: Report of Two Cases*

P. Strohm*, M. V. Neumann, D. Dovi-Akue, K. Kuminack, A. El Tayeh

Klinik für Orthopädie und Unfallchirurgie, Universitätsklinikum, Freiburg, Deutschland

***Question*** Congenital pseudarthrosis of the tibia (CPT) is a rare pathology with an incidence of one in 150.000 births. The specific etiology remains unclear, an association to neurofibromatosis 1 is evident. Successful treatment of CPT is difficult and associated with a high rate of complications, especially osseous non-union. Different surgical techniques have been described using the Ilizarov fixator, vascularised fibular grafts, intramedullary fixation with interposition of cancellous bone or treatment with periosteal flaps.
We would like to present two cases of CPT, which were successfully managed with BMP-2 and noninvasive, pulsed low intensity ultrasound (Exogen).
***Methodology*** A 7-year-old girl jumped off a trampoline onto her right leg affected with CPT type 1. A fracture occurred in the pseudarthrotic area. Surgical management at the day of accident was resection of the pseudarthrosis, intramedullary stabilisation using ESIN and insertion of BMP-2. 6 months later complete osseous healing of the pseudarthrosis and fracture was proven and hard metal removal could be performed.
***Result*** The second case is about an 11-year old boy affected with CPT type 4 to his left leg. After two, failed fibula grafts, a third vascularised fibula graft from the contralateral side was interposed this time adding BMP-2 to the graft–bone connecting area. Additional stabilisation was achieved by placement of an unilateral, external fixator. 6 months later the fixator loosened, the bony mass of the fibula graft was reduced, but the contact area between the graft and bone showed complete osseous healing. Another surgical revision was performed, now using a Duval nail and interposition of a new ipsilateral fibula graft. Postoperatively noninvasive, pulsed low intensity ultrasound was used. After 12 months a residual pseudarthrosis was remaining, otherwise complete osseous healing was documented. The young patient is free of discomfort wearing an additional orthesis. This case is to discuss.

***Conclusion*** Surgical management of the CPT remains challenging. The two presented cases with resection of the pseudarthrosis and local application of BMP-2 show good results. In advanced cases of CPT therapy and treatment appear more difficult. Potentially the application of BMP-2 in the early stages of treatment is medically sensible. The additional use of noninvasive, pulsed low intensity ultrasound should be considered more often. Further investigations in a greater patient cohort are necessary, but first results are promising.

***Poster session P.45***

### *Treatment of Severe Infantile Blount Disease (Langenskiöld Type 5) in a 5-Year-Old Female: A Case Report*

V. Gattung*, R. Hofbauer, M. Poschmann, P. Bernius

Schön Klinikum München Harlaching, Zentrum für Kinder- und Neuroorthopädie, München, Deutschland

***Question*** Blount disease is a developmental disorder of the lower leg leading to a multiplanar deformity with varus, internal rotation and antecurvation of the tibia. In literature several methods of treatment are described, as bracing [2] lateral hemiepiphysiodesis [1], elevation of the medial tibial plateau [4] or combinations of these [3, 5].

There is no clinical evidence for an advantage of one of the mentioned methods, and different treatment options are discussed controversially.

***Methodology*** We treated a 5-year-old caucasian female patient with normal body weight who presented with a varus deformity of 15 degrees and lateral shift of the left lower leg. The patient complained about pain in the left knee during walking and at rest. X-rays and MRI confirmed Blount disease (Langenskiöld Type 5) with hypoplasia of the medial tibial plateau.

Treatment was performed with lateral tibial tension band plating. Because of pain during walking, we additionally applied a pronation base for the shoe.

***Result*** After 1 year follow-up, X-rays revealed an almost physiological mechanical axis. However, the medial proximal tibial plateau is still slipped.

Pain has disappeared completely after 13 months, and the patient was able to walk normally.

Recently performed X-ray showed a slight blount disease of the contralateral right leg, which was also treated with lateral tibial tension band plating.

***Conclusion*** In this case of a severe infantile blount disease in a 5-year-old female, almost normal mechanical axis of the leg was achieved only by growth guidance with a banding plate at the lateral proximal tibia. It has to be considered that the patient was quite young at the point of treatment and body weight was normal.

It remains to be seen, if in future further operations will be necessary, or if the medial tibial plateau will show further remodelling.

***Poster session P.46***

### *Treatment of Steroid-Induced Avascular Osteonecrosis Through Core Decompression and Application of Mesenchymal Stromal Cells*

M. Götze*[1], M. Döring[2], K. Mörth[2], F. Traub[1], T. Kluba[1]

[1]Universitätsklinik Tübingen, Orthopädie, Tübingen, Deutschland, [2]Universitätsklinik Tübingen, Pädiatrie, Tübingen, Deutschland

***Question*** Avascular osteonecrosis (AVN) show a strong association with corticosteroid treatment in autoimmune and hematological diseases. The most commonly affected sites are the femoral head and the knee. Steroids seem to play a major role in inducing AVN, although the particular inducing mechanisms are not known. Decreased perfusion, malfunction of endothelial cells as well as coagulopathies are discussed as possible causes. If the joint surface remains unaffected by the AVN, core decompression is the treatment of choice. Due to their differentiation potential, the easy accessibility and their ability to proliferate quickly, autologous mesenchymal stromal cells (MSC) are a good option to advance the healing process of AVN when used in addition to the core decompression.
***Methodology*** Between 2006 und 2013 we treated 12 children and young adults (sites n = 17) suffering an AVN following therapy with cytostatics, immunosuppressives and corticosteroids with core decompression and additional application of autologous MSCs. The patients were examined clinically and radiologically with a follow-up of 6 years after surgery (range 1–9 years). Criteria for a successful treatment were the clinical development regarding pain at rest and on weight-bearing (visual analog scale), subjective well-being, required follow-on surgery like joint replacement and the radiologic outcome compared to the initial images.
***Result*** More than half of the patients showed radiologically regressive necrotic areas after the treatment, more than 3/4 of the patients experienced a clinical and subjective improvement of their discomfort. Outcome was not dependent on the applied total dose of MSCs (in millions). 1 Patient had to undergo joint replacement surgery due to a worsening of the AVN despite the treatment. There were no negative side effects noted, that could have been traced back to the MSC application.
***Conclusion*** The application of MSCs in AVN treatment is possible and primarily safe. The treatment regimen of core decompression and MSC application can improve the radiologic and clinical outcome of an AVN. The 12 present trials showed, that the use of MSCs is a important addition to the classic core decompression treatment of the AVN.

***Poster session P.47***

### *Unilateral Idiopathic Massive Hypertrophy of Intrinsic Foot Muscles*

M. Keim, C. U. Dussa, C. Multerer, L. Doederlein*

Orthopädische Kinderklinik, Kinderorthopädie, Aschau, Deutschland

***Question*** Introduction: Idiopathic hypertrophy of the intrinsic foot muscles is a rare disorder which can lead to significant stance problems due to a loss of heel and forefoot contact with the ground. The patient walks less stable over cushion like soft plantar mass.
Case presentation: We present two boys (3 and 7 years old) each with a unilateral massive plantar soft tissue swelling that prevents the affected feet from a plantigrade position. The clinical picture resembles a rocker-bottom-foot and examination reveals an elastic subcutaneous painless tumor which covers the medial an plantar region of the sole. and prevents the heel and forefoot from weight-bearing.Weightbearing X-Rays in 2 planes of both feet do not reveal any osseous pathologies or deformities, especially no planovalgus deformity. MRI-scans show a massive hypertrophy of the intrinsic plantar muscles especially of the abductor hallucis and the short hallux flexors. No further abnormalities could be detected.
Question: Beside the problems with shoe fitting and an instability during barefoot walking no further pathologies could be found. Nevertheless the finding is of considerable importance because there is no information concerning further evolution with growth.
We would like to ask if any and if yes which treatment options could be of value. Muscular bulk resection carries the risk of large scarring and neurovascular damage, botzulinumtoxion or other denervating procedures seem to be connected with the risk of recurrence. So there is still the question what could be done to create two rather symmetric feet.

***Poster session P.48***

### *Valgusdeformity and Malalignement oft the Ankle Caused by Multiple Enchondroma in a Child Suffering From M. Ollier*

C. Sippel*, B.-C. Vehse

DRK-Kinderklinik Siegen, Kinderorthopädie, Siegen, Deutschland

***Question*** Medical history: *** 9 year old boy presents initially with massive distension oft he proximal fibula unknown dignity.
*** Caused by the unknown dignity an open histologic veryfication of an enchondroma and the diagnosis M. Ollier was made.
*** Shortening of the fibula with progressive valgusdeformity oft he right ankle caused by the enchondroma occured.
*** Hemiepiphyseodesis oft the distal medial tibiaephysis was done to correct the deformity without complete success.
*** Boy is suffering from pain after sporting activity in the ankle in spite of wearing a stabilisating orthosis.
X-ray: *** in spite oft he hemiepiphyseodesis valgus-malalignement of the ankle joint of 20° in the frontal plane
*** Shortening of the Fibula of 1.5 cm with distension of the distal and proximal fibula caused by the enchondroma.
*** Antecurvation oft he tibia with slightly malalignement of the distal tibial ephysis in the sagittal plane
***Questions*** In the period of growing the boy is well fitted with a dynamic ankle foot orthosis
*** would it be the right way to do the correction with closed wedge osteotomy and lengthening of the fibula after end of growth?
*** would it be better to have an earlier operative treatment because oft the deformity progress?

***Poster session P.49***

### *Vanishing Bone Disease of the Right Lower Leg*

T. Löhle*, T. Wirth

Olgahospital, Orthopädische Klinik, Stuttgart, Deutschland

***Question*** Follow-up on a “How to treat case” presented in 2009.
***Methodology*** The first patient contact with the at that time 11 years old girl with a vanishing bone disease of the right lower leg resulting in a serious bone instability with valgus deformity was in 2007. In addition, there was a deformity of the distal femur. The MRI had shown a massive arterio-venous vessel malformation. The treatment, which was established at that time, consisted of several alcohol/lipiodol intravenous injections under tourniquet with 6 weeks intervals lasting from October 2007 until February 2009. In total, 13 injections were done. This treatment resulted in a reduction of the arterio-venous vessel malformation, however, bony reconstitution did not occur.

***Result*** After having stabilized the vascular situation, we decided on stabilizing the bony part by performing a resection of the area of non-union, intramedullary nailing of both, tibia and fibula plus temporary external fixation from June to December 2010. A complete bony healing could be observed. After having removed the external fixation and cast immobilization for another 10 weeks, the mobilization in below-knee orthosis could be done.

***Conclusion*** Now the patient can independently walk with a shorter right leg using the orthosis and a shoe raise. She did so far refuse to undergo a leg lengthening procedure, which was suggested to her.

Open Access

This article is distributed under the terms of the Creative Commons Attribution 4.0 International License (http://creativecommons.org/licenses/by/4.0/), which permits unrestricted use, distribution, and reproduction in any medium, provided you give appropriate credit to the original author(s) and the source, provide a link to the Creative Commons license, and indicate if changes were made.

